# p21 Regulates Wnt-Notch balance *via* DREAM/MMB/Rb-E2F1 and maintains intestinal stem cell homeostasis

**DOI:** 10.1038/s41420-024-02192-z

**Published:** 2024-09-28

**Authors:** Liangxia Jiang, Jie Tian, Jun Yang, Ronggang Luo, Yongjin Zhang, Chihao Shao, Bing Guo, Xiaoming Wu, Juhua Dan, Ying Luo

**Affiliations:** 1 Department of Pathophysiology, School of Basic Medicine, Guizhou Medical University, Guiyang, Guizhou China; 2 Laboratory of Molecular Genetics of Aging & Tumor, Medical School, Kunming University of Science and Technology, Kunming, Yunnan China

**Keywords:** Stem-cell research, Senescence, Ageing

## Abstract

The crosstalk and balance regulation of Wnt-Notch have been known to be essential for cell fate decision and tissue regeneration, however, how this balance is maintained and how the Wnt-Notch pathways are connected with cell cycle regulation is still not clear. By analyzing the molecular alterations in mouse model with accelerated aging phenotypes due to loss of p21 function in a Werner syndrome background, we observed that Wnt3 and β-Catenin were down-regulated, while Notch1 and Hes1 were up-regulated. This disruption in Wnt-Notch signaling was accompanied by the loss of intestinal stem cell compartment, increase in Bmi1 positive cells, loss of Olfm4/Lgr5 positive cells, and reduced secretory Paneth cells and goblet cells in the intestinal crypts of p21TKO mice. BrdU incorporation, cleaved caspase 3, and Tunel assay results revealed the fast turnover of intestinal epithelia, which may result in abnormal stem cell mobilization and exhaustion of the stem cell reservoir in the intestinal crypts. We further identified shift of DREAM complex towards MMB complex due to the loss of p21 as the cause for faster turnover of intestinal epithelia. Importantly, we identified the E2F1 as the transcriptional regulator for Notch1, which linked the p21-DREAM/MMB/Rb-E2F1 pathway with Wnt-Notch pathway. The overexpression of p21 rescued the DREAM pathway, as well as the imbalance of Wnt-Notch pathway. In summary, our data identify p21 as an important factor in maintaining sequential mobilization, proliferation, and homeostasis of intestinal stem cells.

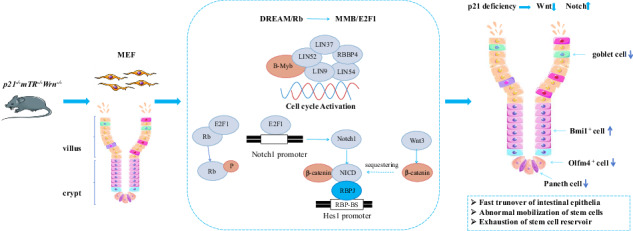

## Introduction

Tissue stem cell maintenance is the basis of tissue renewal, tissue regeneration and repair. The proliferation and differentiation of tissue stem cells is tightly regulated by their respective tissue microenvironment to meet the needs of tissue homeostasis. The dysfunction of any of these mechanisms will lead to aging or damage of tissue stem cells. The aging and damage of tissue stem cells are reflected in alterations in stem cell numbers, shifts between quiescent and proliferating states, and a decline in their ability to proliferate and differentiate, ultimately resulting in tissue dysfunction, aging, and disease [1, 2].

Due to the needs to maintain a higher proliferation potential, compared with somatic cells, stem cells have a unique cell cycle regulation mechanism, represented by a shorter G1 phase of the cell cycle and a rapid entry into S phase, thus resulting in faster cell cycle [3]. However, tissue stem cells need to re-enter the cell cycle frequently from the quiescent state to meet the needs of tissue renewal and repair, and their cell cycle regulation is more complicated [4].

The regulation of cell cycle process is orchestrated by the balance of regulatory mechanisms such as p53-p21-DREAM/MMB/Rb-E2F1 (Drosophila Myb–E2F2/RBF transcriptional repressor complex) pathway [5]. Binding of p21 to cyclin E/CDK1/2 proteins and p16 to cyclin D/CDK4/6 proteins result in inhibition of cyclin/CDK activity and prevent the phosphorylation of Rb family proteins (Rb/Rb1, p130, p107). The activation of Rb family proteins results in transcriptional suppression of downstream targets of E2F1 and MMB, which leads to the inhibition of cell cycle. The DREAM/MMB complex binds to both E2F elements (cell cycle dependent element) on the promoters of G1/S genes, and the cell cycle genes homology region (CHR) on the promoter sites of G2/M genes, while RB-E2F complexes can only interact with E2F sites [6–8]. Studies have shown that the expression of p21 and p16 in embryonic stem cells is limited, and Myc can directly regulate the transcriptional activity of E2F to promote the process of cell cycle [9, 10]. This balance and homeostasis govern cellular stemness, self-renewal, and differentiation, while also preventing tissue and organ aging as well as the development of tumors.

The excessive consumption of stem cells may significantly contribute to the depletion of the stem cell pool in aging tissues. Therefore, regulating the cell cycle to maintain a balance between the quiescent and active proliferative states of stem cells is crucial for preserving the stem cell pool and ensuring effective tissue repair. Studies have shown that the proliferation capacity and absolute number of hematopoietic stem cells increased in p21 knockout mice without injury stimulation. However, when the mice were exposed to the myelotoxic 5-FU, the hematopoietic stem cells of the mice lost their self-renewal ability, resulting in a significant decline in the repair ability of bone marrow injury [11]. Consistent with this, another study found that p21 deletion result in depletion of quiescent stem cells in mouse forebrain tissue, these cells lost their ability to self-renew [12]. These studies suggest that p21 maintains the sequentially regulation of tissue stem cell self-renewal and tissue regeneration by maintaining the resting state of tissue stem cells, however, the molecular mechanism is unclear. They also showed that p21 loss improved small intestine epithelial cell homeostasis and increased hematopoietic stem cell function by improving their ability of hematopoietic reconstruction upon transplantation [13]. This study concluded that knockout of p21 can prolong the lifespan of telomerase-deficient mice without increasing chromosome instability or tumor incidence [13]. In addition, in premature aging mouse models with Pot1b (Protection of Telomeres 1b) knockout, p21 knockout alleviated the senescence phenotype and significantly extended the lifespan of Pot1b knockout mice [14]. Unlike previous studies, these data suggest that p21 loss improves stem cell function and tissue homeostasis. Thus, the role of p21 in stem cell homeostasis and organ aging regulation remains controversial, further data is needed to understand its regulating function.

The Wnt-Notch signaling pathways are among the most important regulatory pathways that drive self-renewal, tissue regeneration, tissue repair, and homeostasis. The Wnt family proteins activate β-Catenin and regulate the transcription of its downstream target genes, such as Lgr5, Axin2, cMyc, cyclin, etc., and participate in the regulation of stem cell division, differentiation, etc. [15]. The Notch proteins and its downstream targets Hes1 determine whether stem cells and early transient amplifying (TA) cells differentiate into small intestinal epithelial cells or secretory cells by balancing Hes1 and Math1 expression [16]. The Wnt and Notch signaling pathways are interconnected and regulate each other [17]. The Wnt-Notch signaling pathway and its related regulatory molecules and the cells producing these factors form a microenvironment (niche) of stem cell self-renewal and tissue regeneration, which regulates the regeneration and repair of a variety of tissues. The balance and crosstalk of Wnt-Notch signaling is essential in regulating small intestinal stem cell homeostasis [18].

In our previous study, we used a mouse model of premature aging syndrome-Werner syndrome (WS) to study the regulatory effects of p21 on aging progress induced by telomere dysfunction. Werner syndrome is a hereditary disease caused by the mutation of Wrn gene. The Wrn protein belongs to family of Recq DNA helicases, and is essential for telomere DNA replication, especially the DNA lagging strand synthesis. The WS mouse with double knocked out of the telomerase RNA components mTerc and Wrn gene (*mTR*^*−/−*^*Wrn*^*−/−*^), faithfully reproduced the phenotypes of human Werner syndrome, such as shortened lifespan, cataract, type II diabetes mellitus, osteoporosis, ischemic heart disease, osteosarcoma, etc. [19, 20]. To study the regulatory effect of p21 on aging caused by telomere damage, the above WS mice (DKO, *mTR*^*−/−*^*Wrn*^*−/−*^) were crossed with p21 knockout (*p21*^*−/−*^) mice, and the G1, G2, and G3 generation of p21TKO mice with progressively shortened telomeres were obtained. In this way, we introduced p21 knockout in the WS background, thus establishing the mouse model of mTR, Wrn, and p21 knockout (p21TKO, *p21*^*−/−*^*mTR*^*−/−*^*Wrn*^*−/−*^) [21].

We were surprised to discover that the absence of p21 did not slow the aging process in WS mice, but rather accelerated it. Specifically, p21G3TKO mice exhibited a significantly reduced lifespan and developed aging-related phenotypes, such as osteoporosis and disrupted intestinal stem cell homeostasis, at an earlier age [21]. Further studies on this basis may further elucidate the regulatory mechanism of p21 on tissue stem cell homeostasis.

The distinctive crypt and villus structure of the small intestine, along with the well-defined distribution of stem cells and proliferating cells, makes it an ideal organ for studying stem cell proliferation and differentiation [22, 23]. Cytokines secreted by mesenchymal cells, Paneth cells and immune cells in the small intestine crypt are involved in the regulation of the crypt microenvironment signal transduction pathway, such as Wnt, Notch, Hh, BMP, etc., thus regulating the homeostasis of small intestine stem cells [22, 24].

In this study, due to its availability and stability, we collect mouse liver tissue to perform RNA-sequencing. Our results from comparative analyses of the RNA-seq data of DKO and p21TKO mouse liver revealed that the Wnt signaling pathway was down-regulated while the Notch pathway was up-regulated. Due to the hierarchy of stem cell structure, we choose small intestine to verify the RNA-seq data. We validated the dysfunctional intestinal stem cell homeostasis, and confirmed the down-regulation of the Wnt pathway and up-regulation of the Notch pathway, as identified by RNA-seq, which may contribute to this dysfunction. Further investigation revealed that the loss of p21 leads to the transition of the DREAM/Rb complex to the MMB/E2F1 complex, resulting in E2F1-mediated transcription of Notch1. This shift disrupts the regulatory balance between the Notch and Wnt pathways. Furthermore, overexpression of p21 can rescue this shift. These findings highlight the critical role of p21 in maintaining intestinal stem cell homeostasis by regulating the interplay between the DREAM/MMB/Rb-E2F1 and Wnt-Notch pathways.

## Results

### Loss of p21 function in the background of Werner syndrome resulted in the up-regulation of Notch and down-regulation of Wnt pathways

**Fig. 1:**
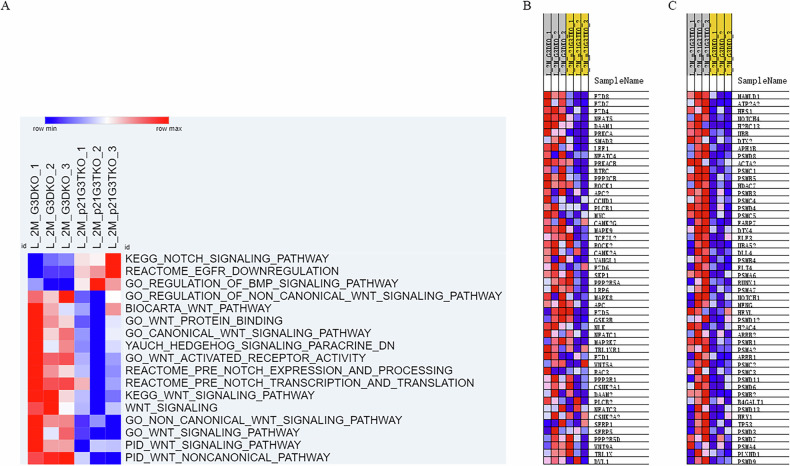
RNA-seq data revealed the up-regulation of Notch and down-regulation of Wnt pathway upon the loss of p21 function in the background of Werner syndrome. **A** The RNA-seq data showing the up-regulated Notch pathway and down-regulated Wnt pathway in p21G2TKO mouse tissue. **B** The top 50 down-regulated Wnt pathway genes in p21G2TKO mouse tissue revealed by GSEA analysis. **C** The top 50 up-regulated Notch pathway genes in p21G2TKO mouse tissue revealed by GSEA analysis.

Previously we discovered that the deletion of p21 in the background of Werner syndrome did not rescue the aging phenotypes, on the contrary, it accelerated the aging progress and caused early onset of progeroid symptoms, such as exhaustion of stem cell reservoir [21]. To understand the molecular pathways altered after p21 deletion, we performed RNA-sequencing on tissues isolated from 3rd generation TKO mice (G3 p21TKO, *p21*^*−/−*^*mTR*^*−/−*^*Wrn*^*−/−*^), which already showed progeroid symptoms. The G3 DKO mice (*mTR*^*−/−*^*Wrn*^*−/−*^) were used as control. Due to its availability and stability, we collect mouse liver tissue to perform RNA-seq. The RNA-seq data was analyzed by ssGSEA (single sample gene set enrichment analysis), by this way, we could disclose the differentially regulated pathways in response to the loss of p21 function (Fig. 1A). The ssGSEA analysis revealed that the Notch pathway was up-regulated in the liver tissue of 2-month-old p21G3TKO mice compared to the same age G3DKO mice. On the other hand, the Wnt pathway was down-regulated in the liver tissue of p21G3TKO mice compared to the G3DKO mice (Fig. 1A). To further investigate the genes involved in these pathways, we performed GSEA (gene set enrichment analysis) using gene sets related to the Notch and Wnt pathways. The top 50 down-regulated Wnt pathway genes in p21G3TKO mouse tissue are displayed in Fig. 1B. Among these, we identified several key components, including the receptor of Wnt pathway-FZD family genes and Lrp6 gene, the Wnt signal transduction factor-DVL gene, the cofactor for the transcription activated by Wnt pathway-Lef1 gene, and the Wnt pathway’s transcriptional product Myc. The Wnt5A and Wnt9A genes were also found down-regulated. Interestingly, we found that the negative regulator of Wnt pathway-Apc gene was down-regulated in p21G3TKO mouse tissue. (Fig. 1B). The top 50 up-regulated Notch pathway genes in p21G3TKO mouse tissue were plotted in Fig. 1C. Among these, the Notch4, Notch1 genes were up-regulated. Other than these, we also found the receptor of Notch pathway-Dll gene, the transcription product of Notch pathway-Hes1 and Hey1 gene, were up-regulated upon the loss of p21 function (Fig. 1C). These data suggested that the loss of p21 resulted in the altered Wnt and Notch signaling, which might lead to the alteration of tissue homeostasis.

**Fig. 2:**
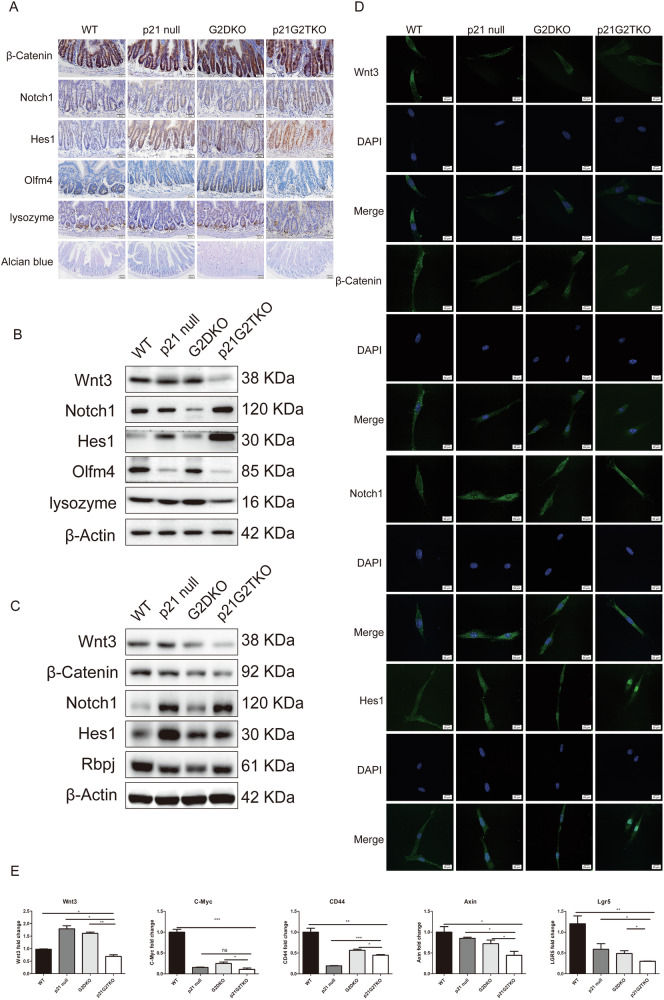
The Notch pathway was up-regulated and the Wnt pathway was down-regulated in the intestinal crypts and MEFs of p21G2TKO mice. **A** The immunohistochemistry staining indicates the up-regulation of Notch and down-regulation of Wnt pathways, along with the reduction of CBC stem cells (Olfm4), Paneth cells (lysozyme), and goblet cells (Alcian blue) in the intestine villi of p21G2TKO mice. **B** Western blotting of the proteins extracted from isolated crypts of four genotypes of mice confirm the up-regulation of Notch and down-regulation of Wnt signaling, along with the reduction of Olfm4 and lysozyme in p21G2TKO crypts. **C** Western blotting of the proteins extracted from MEFs further confirmed the up-regulation of Notch and down-regulation of Wnt signaling in p21G2TKO MEFs. **D** Immunofluorescence staining reveal down-regulation of Wnt3 and β-Catenin, while the up-regulation and the nuclear translocation of Notch1 and Hes1 in p21G2TKO MEFs. **E** The qPCR analysis the RNA from the isolated crypts further confirmed the down-regulation of Wnt3 and its downstream C-Myc, CD44, Axin genes in p21G2TKO crypts, along with CBC markers Lgr5 gene. *N* = 3, ns, no significance; *, *p* < 0.05; **, *p* < 0.01; ***, *p* < 0.001; ****, *p* < 0.0001.

Given the distinct crypt-villus structure and the well-defined hierarchy of stem cells and proliferating cells in the small intestine, we selected this tissue to validate the RNA-seq data and investigate tissue stem cell homeostasis. We investigated the differential expression of the key proteins in Wnt-Notch pathways in small intestine compartments isolated from mice across the four genotypes: the wild type (WT), the p21 single knock out (p21 null, *p21*^*−/−*^), the 2nd generation of WS double knock out (G2DKO, *mTR*^*−/−*^*Wrn*^*−/−*^), the 2nd generation of p21 and WS triple knock out (p21G2TKO, *p21*^*−/−*^*mTR*^*−/−*^*Wrn*^*−/−*^). Additionally, the mouse embryo fibroblasts (MEFs) were established from these four genotypes of mice for cell based experiments. The immunohistochemistry staining of the intestinal villi illustrated that, compared to the other three genotype of mice, the β-Catenin was down-regulated in majority of the crypt cells of p21G2TKO mouse, displaying a punctuate staining pattern (Fig. 2A, β-Catenin). In contrast, Notch1 and Hes1 were up-regulated and localized in the nuclei of crypt cells in p21G2TKO mice (Fig. 2A, Notch1 and Hes1). When we found this altered expression of the key proteins in Wnt-Notch pathways, we were wondering whether the depletion of Lgr5^+^ stem cells we observed earlier [21] was due to this imbalance between Wnt and Notch signaling. To further understand the cell linage change that occurred upon loss of p21 function, we first performed immunohistochemistry staining of the intestinal villi for Olfm4, the marker for crypt base columnar stem cell (CBC). Our results showed that the abundance of Olfm4 positive cells was indeed reduced in the crypts of p21G2TKO mice (Fig. 2A, Olfm4). Since the Paneth cells are known to be essential in secreting Wnt proteins and forming CBC maintenance niche, we then investigated the secretory cell linage by staining the Paneth cells and goblet cells with lysozyme and Alcian blue, respectively. The results revealed that abundance of both Paneth cells and goblet cells decreased in the crypts of p21G2TKO mice, compared with WT, p21 null, and G2DKO mice (Fig. 2A, lysozyme, Alcian blue). The reduction of Paneth cells further supports the loss of CBC in the crypts of p21G2TKO mice.

Since the crypts are the key compartments for CBC stem cells, we isolated the crypts from the intestine of four genotypes of mice and extracted the crypt proteins for Western blot. The results revealed that Wnt3 was down-regulated, while Notch1 and Hes1 were up-regulated in the crypts of p21G2TKO mice, compared with WT, p21 null, and G2DKO mice (Fig. 2B, Wnt3, Notch1, Hes1). Consistent with the tissue immunohistochemistry results, the Olfm4 was down-regulated in the crypts of both p21 null and p21G2TKO mice compared with WT and G2DKO mice (Fig. 2B, Olfm4). The lysozyme was down-regulated in the isolated p21G2TKO crypts, confirming the decrease of Paneth cells abundance in the crypts of p21G2TKO mice (Fig. 2B, lysozyme).

To further confirm these results, we prepared MEFs from the above four genotypes of mice. Western blot data, consistent with the tissue data, revealed that Wnt and β-Catenin were down-regulated in p21G2TKO compared to G2DKO MEFs, while Notch1, Hes1, and Rbpj were up-regulated in both p21 null and p21G2TKO compared to G2DKO MEFs (Fig. 2C). By immunofluorescence staining, we found that Wnt3 and β-Catenin were down-regulated in p21G2TKO MEFs, compared with WT, p21 null, and G2DKO MEFs (Fig. 2D, Wnt3, β-Catenin), while the Notch1 was up-regulated and translocated into the nuclei of p21G2TKO MEFs (Fig. 2D, Notch1). Interestingly, the Notch1 downstream key protein Hes1 was up-regulated in p21G2TKO MEFs, and translocated into nuclei of the cells, suggesting the enhancement of its function in transcription regulation (Fig. 2D, Hes1).

We then extracted the RNA from the crypts and performed RT-qPCR to compare the expression level of Wnt genes. The results revealed that Wnt3 and its downstream C-Myc, CD44, Axin genes were down-regulated in isolated p21G2TKO crypts, compared with WT and G2DKO crypts (Fig. 2E). The CBC markers Lgr5 gene was also down-regulated in isolated p21G2TKO crypts, compared with WT and G2DKO crypts (Fig. 2E).

Collectively, these findings confirmed the loss of CBC stem cells and Paneth cells in the intestines of p21G2TKO mice compared to WT, p21 null, and G2DKO mice. This suggests an alteration in intestinal stem cell differentiation and cell lineages, potentially leading to the depletion of the stem cell reservoir, likely due to changes in the Wnt-Notch pathways following p21 loss.

### The shift of DREAM/Rb to MMB/E2F1 pathway promote cell proliferation, cell cycle progression, and stem cell mobilization upon the loss of p21 function

**Fig. 3:**
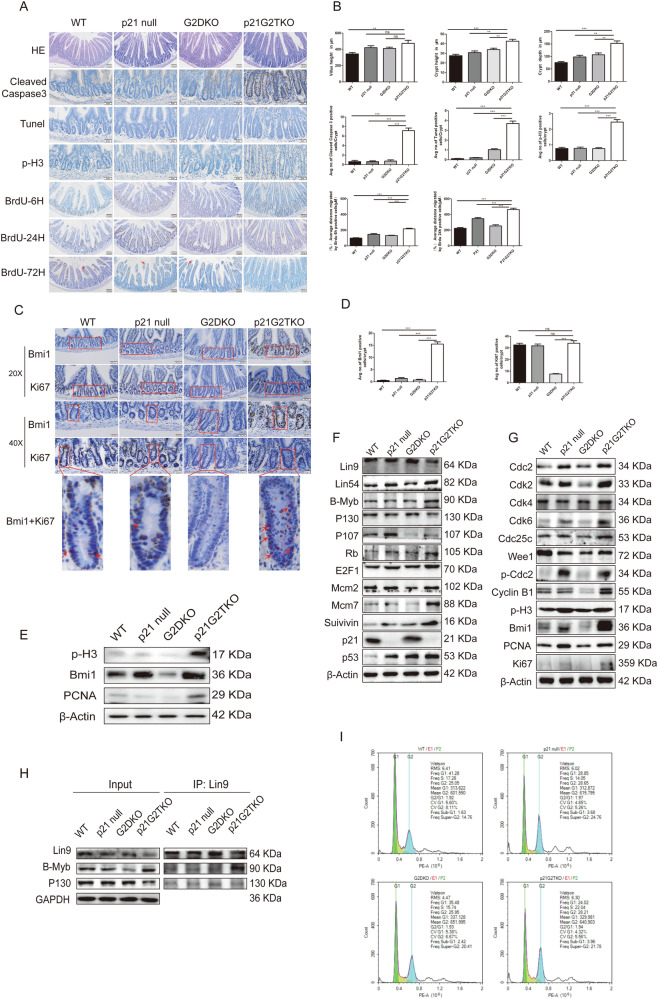
The shift of DREAM/Rb to MMB/E2F1 pathway promoted the cell proliferation, cell cycle progression, and stem cell mobilization in p21G2TKO mice. **A** The comparison of morphology (H&E), cell apoptosis (cleaved caspase3, Tunel), cell proliferation (phosphorylated H3 and BrdU), and mobilization (BrdU) occurring in the intestine villi of different genotype of mice reveal the fast turnover of intestinal epithelia in p21G2TKO mice. **B** The quantification of (**A**). *N* = 3, ns, no significance; **, *p* < 0.01; ***, *p* < 0.001. **C** The immunohistochemistry staining reveals a robust increase of Bmi1^+^ cells (+4 stem cell marker) in the crypts of p21G2TKO mice, which partially overlapped with the proliferation marker Ki67 by staining in serial sections. The red frames show the similar structures across serial sections, and the red arrows point to colocalization. **D** The quantification of (**C**). *N* = 3, ns, no significance; ***, *p* < 0.001. **E** The Western blotting of isolated crypt protein extract indicate an increased expression of Bmi1 in both p21 null and p21G2TKO (inverse expression pattern with Olfm4 in Fig. 2B), while the phosphorylated H3 and PCNA were only up-regulated in p21G2TKO crypts. **F** The Western blotting of proteins from MEFs indicate the expression of proteins favors the MMB/E2F1 complex than the DREAM/Rb complex in p21G2TKO cells. **G** The Western blotting of proteins from MEFs indicate the down-stream proteins of DREAM/MMB/Rb-E2F1 pathway is shifted towards cell cycle promoting form (MMB/E2F1) in p21G2TKO cells. **H** Co-IP confirm that the binding pattern of DREAM/MMB/Rb-E2F1 complex shifted towards the active form for cell cycle progression in p21G2TKO cells. **I** The cell cycle analysis reveals that p21G2TKO cells contained lesser percentage of G1 phase cells and higher percentage of S phase cells.

Other than the impact of altered Wnt-Notch signaling, given the well-known function of p21 in inhibition of cell cycle progression, we suspected that the CBC exhaustion could be the consequence of over mobilization of quiescent stem cells *via* cellular proliferation upon p21 loss, especially in the background of Werner syndrome. To test this hypothesis, we analyzed the cell migration, proliferation, and apoptosis status along the various niches across small intestine villi. The hematoxylin-eosin (H&E) staining results revealed that the average villus height did not differ significantly between G2DKO and p21G2TKO mice (Fig. 3A, quantified in B). However, both the average crypt height and depth were increased in p21G2TKO mice compared to G2DKO mice (Fig. 3A, quantified in B), suggesting an enlargement of the crypts in p21G2TKO mice. Interestingly, both cleaved capspase3 staining and Tunel staining revealed a higher rate of cellular apoptosis in the small intestine villi of p21G2TKO mice compared to other genotypes (Fig. 3A and quantification in B). While at the same time, the cell proliferation rate is also increased as shown by phosphorylated H3, a marker for cells in M phase (Fig. 3A and quantification in B). The high rate of cell apoptosis and proliferation suggested a much faster turnover of cells in the villi of p21G2TKO mice. To verify this, we then traced the cell migration along the villi of these four genotypes of mice by BrdU incorporation. The results revealed that at 6 h after BrdU injection, the BrdU signal was retained mostly in the crypt cells of all four genotypes of mice (Fig. 3A, 6 H and quantification in 3B, some BrdU labeled cells located in the middle of villi matrix were disregarded as they do not belong to intestinal epithelia). While at 24 h after BrdU injection, a lot of BrdU labeled cells migrated along the villi could still be observed in p21G2TKO and p21 null mice, while most BrdU labeled cells still retained in the crypts of WT and G2DKO mice (Fig. 3A, 24 H), the migration distance was significantly different (Fig. 3B, 24 H). At 72 h after BrdU injection, the BrdU labeled cells were no longer found in the villi epithelia of p21G2TKO (Fig. 3A, 72 H), while we still could observe BrdU labeled epithelia at the tip part of villi in WT, p21 null, and G2DKO mice (Fig. 3A, 72 H, red arrow pointed cells). Together these data support the fast migration and turnover of cells in the villi of p21G2TKO mice, probably due to increased cell proliferation, as well as apoptosis.

Given this fast turnover of cells, we were wondering what would happen to the +4 stem cells (position 4 stem cells, zoned between CSC and transient amplification (TA) cells) in the crypts. Since Bmi1 is a marker for +4 stem cells, we stained crypts for Bmi1 to visualize these cells. Surprisingly, our data showed a reduction of Bmi1 expressing cells in the crypts of G2DKO mice compared with WT and p21 null mice, while increased Bmi1 expressing cells in the crypts of p21G2TKO mice (Fig. 3C, Bmi1). This increase in Bmi1-positive cells in the crypts of p21G2TKO mice was further confirmed by immunofluorescence staining (Supplementary Fig. 1). If Bmi1 was an exclusive marker for +4 stem cells, these data would suggest an obvious increase of +4 stem cells in the intestine of p21G2TKO mice. However, it is more likely that these data indicate an increase in transient amplification cells in the intestines of p21G2TKO mice, as Bmi1 can also serve as a marker for proliferating cells. To test this hypothesis, we compared the staining patterns of proliferation marker Ki67 and Bmi1 by performing immunohistochemistry staining on serial sections, aiming to find similar structures and merge the Ki67 and Bmi1 signals. The results showed that in the crypts of WT, p21 null, and G2DKO mice, the Ki67 positive cells were more abundant than the Bmi1 positive cells (Fig. 3C, 20X and 40X, with red frames indicating similar structures in serial sections. The signals were quantified in D). However, in the crypts of p21G2TKO mice, where Bmi1 signals were robust, the number of Ki67-positive cells matched that of Bmi1-positive cells (Fig. 3C, 20X and 40X, with red frames indicating similar structures in serial sections. Signals were quantified in D). To further merge these two signals, we select a single crypt to compare the signals of Bmi1 and Ki67. The results revealed multiple colocalization of Bmi1 and Ki67 in a crypt of p21G2TKO mice, whereas very few or none was found in the crypts of WT, p21 null, and G2DKO mice (Fig. 3C, Bmi1 + Ki67, red arrow pointed to the colocalization). These data indicated the significant increase of proliferative cells in the crypts of p21G2TKO mice, and these cells were Bmi1 positive.

We further analyzed Bmi1 expression using isolated crypt protein extracts from the four mouse genotypes. The results confirmed increased Bmi1 protein levels in both p21 null and p21G2TKO mice compared to WT and G2DKO mice (Fig. 3E). Interestingly, when we aligned these findings with the expression pattern of Olfm4 in Fig. 2B, we observed that Olfm4 and Bmi1 were inversely regulated in p21 null and p21G2TKO crypts, i.e., when Olfm4 was down-regulated, Bmi1 was up-regulated (Fig. 2B and Fig. 3E). Additionally, proliferative markers, such as the phosphorylated H3 and PCNA, were only up-regulated in p21G2TKO crypts (Fig. 3E). Together these data suggest that the p21 loss itself already resulted in the switch of Olfm4^+^ cells to Bmi1^+^ cells in the crypts, while only in p21G2TKO background, the cells were highly proliferative.

It is well known that p21 regulates DREAM/MMB/Rb-E2F1 pathway (DREAM/Rb as the suppressive form, and MMB/E2F1 the activated form) and inhibits cell cycle progression [8], we investigated whether the proteins in DREAM/MMB/Rb-E2F1 complex and their binding partner proteins play any role in this process. We used the MEFs from the four mouse genotypes to perform Western blot and co-immunoprecipitation (Co-IP). We found that the core protein of DREAM/MMB pathway Lin9 was slightly down-regulated in p21G2TKO cells, while Lin54 was up-regulated compared with G2DKO cells (Fig. 3F). Additionally, the activating protein B-Myb and E2F1 were up-regulated, while the inhibitory protein p130 were down-regulated in p21G2TKO cells compared with G2DKO cells (Fig. 3F). Thus, although the inhibitory protein p107 and Rb were slightly up-regulated in p21G2TKO cells compared with G2DKO cells, the up-regulation of down-stream target proteins Mcm2, Mcm7, Survivin indicated the DREAM/MMB/Rb-E2F1 pathway was shifted to cell cycle promoting form in p21G2TKO cells compared with G2DKO cells (Fig. 3F). The up-regulation of cell cycle proteins PCNA, Ki67, phosphorylated H3, Cdc2, phosphorylated Cdc2, Cdk2, Cdk4, Cdk6, Cdc25C, Cyclin B1, and Wee1 indicated the enhancement of the cell cycle progression (Fig. 3G). Here, we again observed the up-regulation of Bmi1 in p21 null and p21G2TKO cells (Fig. 3G). By Co-IP, we found that the core protein Lin9 bound more B-Myb and less p130 in p21G2TKO cells compared with G2DKO, indicated the DREAM/MMB/Rb-E2F1 complex shifted to the active form for cell cycle progression in p21G2TKO cells (Fig. 3H). Finally, the cell cycle analysis also revealed that p21G2TKO cells contained less percentage of G1 phase cells and more percentage of S phase cells (Fig. 3I).

Together these data suggest that the loss of p21 in the G2TKO background (p21G2TKO) promotes the active form of the DREAM/MMB/Rb-E2F1 complex to drive cell cycle progression, which in turn accelerate the turnover of intestine villi epithelia cells and impact the stem cell mobilization.

### Upon the loss of p21 function, activated E2F1 up-regulated the transcription of Notch1, resulting in the activation of Notch pathway, and inhibition of Wnt pathway

Given the alteration of both Wnt-Notch, DREAM/MMB/Rb-E2F1 pathways, our next question was whether p21 loss affects Wnt-Notch pathway *via* DREAM/MMB/Rb-E2F1. Since the DREAM/MMB/Rb-E2F1 complex regulates its down-stream proteins by transcriptional regulation, we investigated whether this mechanism underlies the imbalance in the Wnt-Notch pathway.

**Fig. 4:**
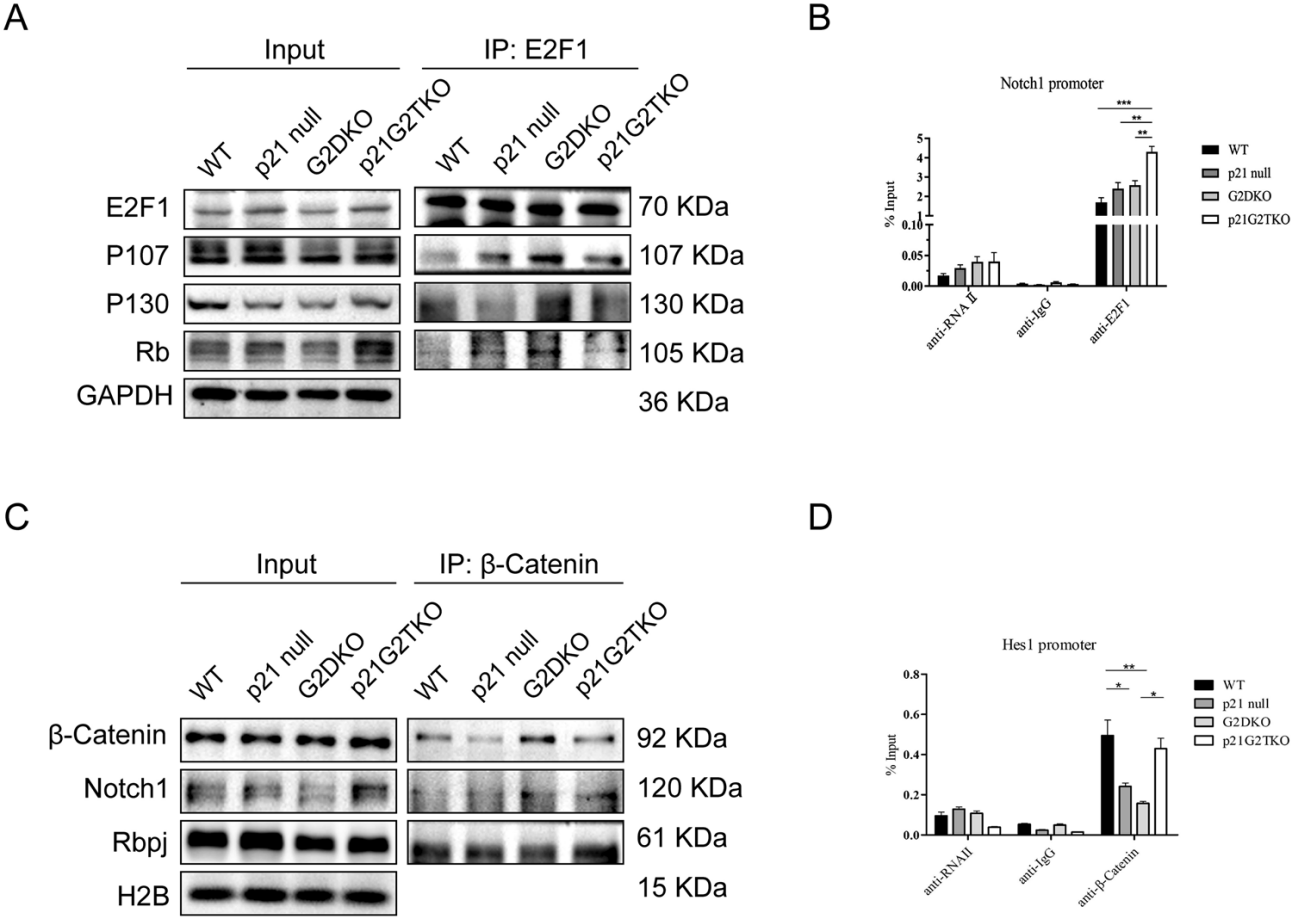
The activated E2F1 up-regulated the transcription of Notch1, resulted in the activation of Notch pathway, and inhibition of Wnt pathway in p21G2TKO MEFs. **A** Co-IP with anti-E2F1 antibody indicate that E2F1 binding Rb reduced in p21G2TKO cells. **B** ChIP assay with anti-E2F1 antibody reveals that E2F1 binds Notch1 promoter with higher efficiency in p21G2TKO cells, suggesting the released E2F1 activity promoted Notch1 transcription. *N* = 3, **, *p* < 0.01; ***, *p* < 0.001. **C** Co-IP with anti-β-Catenin antibody indicated more Notch1 and Rbpj proteins are bound to β-Catenin in p21G2TKO cells, suggesting that the up-regulation of Notch1 results in its enhanced binding to β-Catenin. **D** ChIP assay with anti-β-Catenin antibody showed that more β-Catenin bound to Hes1 promoter in p21G2TKO cells. *N* = 3, *, *p* < 0.05; **, *p* < 0.01.

We first performed Co-IP in MEFs from the four genotypes using an anti-E2F1 antibody to assess changes in E2F1 binding pattern upon the loss of p21. The data showed that both E2F1 and Rb protein levels slightly elevated in p21G2TKO cells, however, the level of E2F1 binding to Rb was reduced compared to G2TKO cells (Fig. 4A). Interestingly, we also observed reduced binding of p107 to E2F1 in p21G2TKO cells (Fig. 4A). To test whether the released E2F1 contributed to Notch1 transcription, we performed the ChIP assay with an anti-E2F1 antibody. We found significantly more E2F1 bound to the promoter of Notch1 in p21G2TKO cells compared to the other three genotypes (Fig. 4B), suggesting that the released E2F1 activity promote Notch1 transcription, lead to increased Notch1 protein level in p21G2TKO cells. It has been shown that Notch1 could bind to β-Catenin and inhibit Wnt signaling. We then investigated the binding of Notch1 to β-Catenin, in the four genotypes of cells by Co-IP. The results indicated that more Notch1 and Rbpj proteins bound to β-Catenin in p21G2TKO cells compared to other three genotype of cells (Fig. 4C), suggesting that the up-regulated Notch1 in p21G2TKO cells enhanced its binding to β-Catenin, which might then enhance transcription of Notch pathway and effectively sequester the β-Catenin from Wnt pathway. To verify this, we then conducted the ChIP assay using an anti-β-Catenin antibody. The data showed more β-Catenin bound to the Hes1 promoter in p21G2TKO cells compared to G2TKO cells (Fig. 4D), explaining the up-regulation of Hes1 protein in these cells. Interestingly, we also observed robust binding of β-Catenin to the Hes1 promoter in WT cells (Fig. 4D), which did not result in the up-regulation of Hes1 protein level.

Together these data indicate that the loss of p21 result in the down-regulation of the inhibitory function of the Rb family, which releases E2F1 protein activity. These released E2F1 in complex with β-Catenin bind to Notch1 promoters and thus enhance Notch signaling pathway. This, in turn, might inhibit Wnt pathway signaling by competing for β-Catenin.

### Overexpression of p21 rescued the imbalance of Wnt-Notch, and shifted the DREAM/MMB/Rb-E2F1 complex towards its inhibitory function

**Fig. 5:**
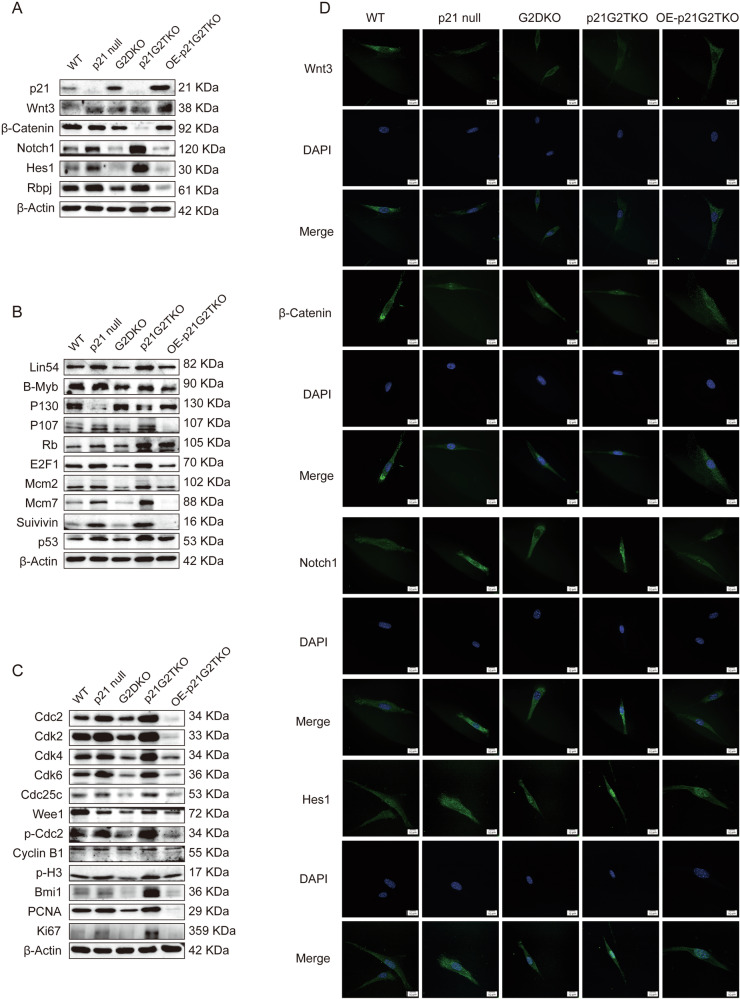
Overexpression of p21 rescued the imbalance of Wnt-Notch, and shifted the DREAM/MMB/Rb-E2F1 complex towards its inhibitory function. **A** Overexpression of p21 in p21G2TKO cells restored the expression of Wnt3, β-Catenin proteins, and suppressed the expression of Nocth1, Hes1, and Rbpj. **B** Overexpression of p21 suppressed the expression of MMB/E2F1 proteins and restored the inhibition DREAM/Rb proteins. **C** The MMB down-stream proteins and cell cycle related proteins were also suppressed by p21 overexpression. **D** Immunofluorescence staining indicated the up-regulation of Wnt3 and β-Catenin, and suppression of Nocth1, Hes1 protein level after overexpression of p21.

To further confirm the role of p21 in regulating the balance of Wnt-Notch pathway, we overexpressed p21 back in the p21G2TKO cells and investigated whether this could rescue the imbalance. By transiently overexpressing p21 in p21G2TKO cells, we successfully restored the expression of Wnt3, β-Catenin proteins, and suppressed the expression of Nocth1, Hes1, and Rbpj (Fig. 5A, OE-p21G2TKO). Additionally, overexpression of p21 in p21G2TKO cells led to shift from MMB/E2F1 to DREAM/Rb activity, as evidenced by the up-regulation of p130 and Rb, down-regulation of Lin54, B-Myb, and E2F1 (Fig. 5B, OE-p21G2TKO). Interestingly, the p107 was down-regulated by p21, the function of this need to be further studied. Consistent with this, the MMB/E2F1 down-stream Mcm2, Mcm7, Survivin were also suppressed by p21 overexpression (Fig. 5B, OE-p21G2TKO). The suppression of cell cycle related proteins Cdc2, phosphorylated Cdc2, Cdk2, Cdk4, Cdk6, Cdc25C, Cyclin B, and Wee1 (Fig. 5C, OE-p21G2TKO), and the down-regulation of phosphorylated H3, Bmi1, PCNA, and Ki67 also indicated the inhibition of the cell cycle progression by p21 (Fig. 5C, OE-p21G2TKO), probably *via* DREAM/MMB/Rb-E2F1.

We further investigated the alteration of Wnt-Notch pathway proteins by immunefluroscence staining. The results confirmed the up-regulation of Wnt3 and β-Catenin, and suppression of Nocth1, Hes1 protein level after overexpression of p21 (Fig. 5D, OE-p21G2TKO).

These findings support the role of p21 in maintaining appropriate levels of Notch1 and Wnt3, which is crucial for balancing the Wnt-Notch pathways. The loss of p21 function leads to an imbalance in the Wnt-Notch pathway, contributing to the disruption of intestinal tissue stem cell homeostasis.

## Discussion

It has been shown that both Wnt and Notch pathways are essential for the stem cell renewal and the balance of Wnt-Notch signaling is important for the proper mobilization, proliferation, and differentiation of stem cells. While how the Wnt-Notch balance contributed to the stemness maintenance is still controversial. A study on prostate stem cells has found that induction of Wnt signaling promoted stem cell proliferation and self-renewal, while Notch signaling inhibits the proliferation of stem cells, suggesting that Wnt and Notch act antagonistic balanced regulatory relationship in the proliferation and differentiation of stem cells [25]. However, it has also been shown that Notch inhibition induced rapid CBC cell loss, with reduced proliferation, apoptotic cell death, and reduced efficiency of organoid initiation [26]. It has been reported that either irradiation-induced tissue damage or expressing active Notch1 could induce a subset of Paneth cells to dedifferentiate, while constitutive activation of the Wnt/β-catenin pathway did not induce dedifferentiation [27]. A study revealed that levels of endoplasmic reticulum (ER) stress and activation of the unfolded protein response are low in stem cells compared to TA cells, the induction of ER stress causes loss of stemness [28]. Using a new flow sorting protocol, the mouse colon epithelial cells have been isolated to study the transcriptomic and proteomic signatures of stemness and differentiation. The data reveal that the RNA processing targets regulators of cell cycle, RNA, cell adhesion, SUMOylation, and Wnt- Notch signaling [29]. These data indicate that the crosstalk and balance between Wnt-Notch are quite complicated, and more studies with different genetic or physiological background are needed.

Also, it is still not clear how Wnt-Nocth pathways connect with cell cycle regulators, such as p53-p21, p16-Rb pathways. It has been shown that p21 dysfunction results in the depletion of stem cell compartment [11, 12, 21]. However, the relationship between the p21-regulated cell cycle pathway and the Wnt-Notch pathway has remained unclear. In this study, we elucidate the mechanisms by which p21 links the Wnt-Notch pathway with the DREAM/MMB/Rb-E2F1 pathway, and explore how this interaction may influence cell cycle regulation and intestinal stem cell homeostasis.

By comparing the molecular alterations in the mouse model with accelerated aging phenotypes due to the loss of p21 function in Werner syndrome background (p21G2TKO) with the mouse model with normal p21 function (G2TKO), we could dissect the function of p21 in Wnt-Notch pathways and stem cell homeostasis. It has been shown that the decline of canonical Wnt signaling in intestinal stem cells leads to decreased stem cell regenerative potential upon aging [30]. Consistent with this study, we observed the down-regulation of Wnt3 and β-Catenin aligned with the loss of intestinal stem cell compartment in p21TKO mice. It has also been shown that ectopic Notch signaling in adult intestinal progenitor cells creates a bias against secretory cell fates, while ectopic Notch activation in the embryonic foregut leads to reversible defects in villus morphogenesis and the loss of the proliferative progenitor compartment [31]. We also observed the up-regulation of Notch1 and Hes1 result in the decrease of secretory Paneth cells and goblet cells. These data suggest the loss of balance of Wnt-Notch signaling and the loss of stem cells, as well as secretive cells in the intestine villi of p21TKO mice. Now the question is where have those stem cell gone? We observed an abnormal increase of Bmi1 positive cells in the intestine villi of p21TKO mice. By overlapping the Bmi1 signals with Ki67 signals, we identified a robust increase of proliferative cells in the small intestine villi of p21TKO mice. It has been demonstrated that upon the loss of Lgr5-expressing cells, the Bmi1-expressing cells serve as an alternative stem cell pool to maintain normal intestinal homeostasis [32, 33]. The existence of an inverse correlation between p53/p21 and the expression of Bmi1 promotes the stem cell self-renewal [34]. While in our case, we did observe the increase of Bmi1 positive cells along with the loss of Olfm4/Lgr5 positive cells, however, the Bmi1 cells could not compensate for the intestinal homeostasis. Instead, the Bmi1 markers, aligned with other proliferative markers, such as Ki67, phosphorylated H3, indicating the increasing of the cell proliferations in the intestine villi of p21TKO mice. The BrdU incorporation, the cleaved caspase 3, and the Tunel assay of cells further revealed the fast turnover of intestinal epithelia, which might exhaust the stem cell reservoir.

Given the downstream target of p21, we further identified the shift of DREAM/Rb complex to MMB/E2F1 complex is responsible for the rapid turnover of intestinal epithelia. Importantly, we identified E2F1 as the transcription regulator of Notch1, thereby linking the p21-DREAM/MMB/Rb-E2F1 pathway with Wnt-Notch pathway. It has been found that increased E2F1 binding to the Notch1 promoter leads to Notch1 upregulation in endothelial cells [35]. Additionally, activated Notch1 (NICD) has been found to bind to β-Catenin, regulating the expression level of Hes1 and sequestering β-Catenin from Wnt pathway [36, 37]. Membrane-bound Notch has also been found to physically associates with β-Catenin in stem and colon cancer cells, negatively regulating the post-translational accumulation of active β-Catenin protein [38]. E2F1 is also recognized as a potent and specific inhibitor of β-catenin/T-cell factor (TCF)-dependent transcription [39]. Interestingly, we observed a high level of β-catenin binding to HES1 promoter in WT cells compared with p21 null cells, despite similar Hes1 protein levels. This raises the possibility that other cofactors in WT cells might prevent active transcription of Hes1.

These findings collectively support that the loss of p21 function results in the shift from the cell cycle inhibitory DREAM/Rb complex to the activating MMB/E2F1 complex, leading to increased E2F1 activity. This shift promotes cell cycle progression in the aging context, facilitating cell proliferation and potentially stem cell mobilization. Concurrently, the up-regulated E2F1 binds to the Notch1 promoter, leading to the up-regulation of Notch1 and its downstream target Hes1. The increased Notch1 proteins suppress Wnt3 signaling by competing for β-Catenin, causing up-regulation of Notch signaling and down-regulation of Wnt signaling, ultimately impacting tissue stem cell homeostasis.

Consistent with our finding, the early study has revealed that Rb family triple knockout (TKO) mice develop a cell-intrinsic myeloproliferation that originates from hyperproliferative early hematopoietic progenitors and is accompanied by increased apoptosis in lymphoid progenitor populations. The presence of a single p107 allele is sufficient to largely rescue these defects [40]. In our case, p21 is further upstream of the Rb family. Interestingly, we have also found that dysfunction of p16 another upstream regulator for Rb family activity, rescues the loss of intestinal stem cell compartment caused by Werner syndrome [21]. Thus, more studies need to be conducted to understand the crosstalk and balances between those pathways.

In summary, p21 may play an important role in maintaining the sequential mobilization, proliferation, and homeostasis of small intestinal tissue stem cells by regulating the balance of Wnt-Notch pathway. It is surprising that a cell cycle inhibitor, p21, known to be one of the markers for aging, could be essential in prevent aging process by maintaining the proper stem cell homeostasis. This highlights the double-edged sword role of p21 in aging progression. While p21 may act as a quality controller for tissue homeostasis, its dysfunction has consequences that are highly dependent on the cellular context. Caution is needed when considering p21 as a target for anti-aging strategies.

## Material and methods

### RNA-sequencing and Bioinformatics analysis

The liver tissue from the 3rd generation TKO mice (p21G3TKO, *p21*^*−/−*^*mTR*^*−/−*^*Wrn*^*−/−*^) and the control G3DKO mice (*mTR*^*−/−*^*Wrn*^*−/−*^) were harvested to isolate total RNA for RNA-sequencing. The RNA-seq data was analyzed by ssGSEA (single sample gene set enrichment analysis) to identify the differentially regulated pathways in response to the loss of p21 function [41]. The C2 gene sets from Molecular Signatures database [42] were used as the pathway database for ssGSEA analysis. The gene set enrichment analysis (GSEA) was applied on RNA-seq data to further analyze the individual gene sets involved in related pathways.

### Mice and MEF cells

All mouse protocols have been approved by the Animal Care and Use Committee of Guizhou Medical University. All mouse strains are on a C57BL/6 background. We crossed the WS mice (double knock out, DKO, *mTR*^*−/−*^*Wrn*^*−/−*^) with p21 null (*p21*^*−/−*^) mice and obtained the first generation (G1) TKO mice p21G1TKO (G1 *p21*^*−/−*^*mTR*^*−/−*^*Wrn*^*−/−*^). The mice were then inbred to get p21G2TKO (G2 *p21*^*−/−*^*mTR*^*−/−*^*Wrn*^*−/−*^) mice. The WT (wild type), p21 null (*p21*^*−/−*^), G2DKO (G2 *mTR*^*−/−*^*Wrn*^*−/−*^) mice were used as controls. All mice were maintained in a specific pathogen-free mouse facility. Adult mice of both sexes aged 2–3 months were randomly collected according to their genotypes and used for experiments. The MEF cells derived from the four genotypes of mice were harvested on e13.5 days and cultured in Dulbecco’s Modified Eagle Medium (DMEM) with 10% fetal bovine serum at 37 °C with 5% CO2 and 3% O2. All experiments with MEFs were performed with cells cultured for less than 5 passages.

### Western blot

Cells were harvested and lysed in RIPA buffer containing protease inhibitor cocktail (Roche, Switzerland). The 20 µg of total protein were separated by SDS-PAGE and then transferred to PVDF membrane. After blocking in 10% non-fat milk for 1 h at room temperature, membranes were incubated with primary antibodies overnight at 4 °C. The membranes were then incubated with horseradish peroxidase-labeled secondary antibodies and visualized with ECL. The Western blot images were captured by Tanon 5200 imager (Tanon Tech, China). For reference, all the original Western blot images are attached as Supplemental Materials. The primary antibodies used were anti-Bmi1 (1:1000, Abcam), anti-B-Myb (1:1000, Abmart), anti-β-Catenin (non-phosphorylated (active), 1:1000, CST), anti-Cdc2 (1:1000, CST), anti-phosphorylated Cdc2 (p-Try15, 1:1000, CST), anti-Cdc25c (1:1000, SAB), anti-Cdk2 (1:1000, CST), anti-Cdk4 (1:1000, Santa Cruz), anti-Cdk6 (1:1000, CST), anti- Cyclin B1 (1:1000, Abcam), anti-E2F1 (1:1000, NOVUS), anti-phosphorylated H3 (p-S10, 1:1000, Abcam), anti-Hes1 (1:1000, CST), anti-Ki67 (1:1000, CST), anti-Lin54 (1:1000, Abcam), anti-Lin9 (1:1000, Proteintech), anti-Lysozyme (1:1000, Abcam), anti-Mcm2 (1:1000, Abcam), anti-Mcm7 (1:1000, Santa Cruz), anti-Notch1 (1:1000, CST), anti-Olfm4 (1:1000, CST), anti-p107 (1:1000, Santa Cruz), anti-p130 (1:1000, Abcam), anti-p21 (1:1000, Santa Cruz), anti-p53 (1:1000, Proteintech), anti-PCNA (1:1000, Abcam), anti-Rb (1:1000, BD), anti-Rbpj (1:1000, CST), anti-Survivin (1:1000, CST), anti-Wee1 (1:1000, Abcam), anti-Wnt3 (1:1000, Abcam), anti-β-actin (1:1000, Santa Cruz), anti-GAPDH (1:5000, GeneTex), anti-H2B (1:1000, Abcam).

### Immunohistochemistry staining

The mouse small intestine tissue was harvested and fixed in 4% paraformaldehyde at 4 °C overnight, then was alcohol-dehydrated and embedded in paraffin. The paraffin-embedded tissue blocks were sliced into 5 µm slices for experiments. For H&E staining, tissue sections were de-paraffinized, rehydrated, and proceeded to H&E staining. For immunohistochemistry staining, tissue sections were de-paraffinized and rehydrated, then treated with 3% hydrogen peroxide to block endogenous peroxidase activity. The antigen was retrieved by boiling in high pressure pot in 10 mM citrate buffer (pH 6). The slides were incubated with 10% goat serum and incubated with primary antibody overnight at 4°C. Secondary antibody was applied for 20 min at 37 °C after treated with secondary antibody enhancement solution for 20 min at 37 °C. After washing three times in 1x PBS, the slides were incubated with 3,3-diaminobenzidin (DAB) at room temperature in the dark. The images were captured by a Leica DMLS microscope (Leica, Germany). About 5 to 6 images were collected for quantitative analysis by ImageJ (USA). The primary antibodies used were anti-Bmi1 (1:500, Abcam), anti-Caspase3 (cleaved, 1:300, CST), anti-Hes1 (1:300, CST), anti-β-Catenin (non-phosphorylated (active), 1:300, CST), anti-phosphorylated H3 (p-S10, 1:500, Abcam), anti-Lysozyme (1:1200, Novus), anti-Notch1 (1:500, CST), anti-Olfm4 (1:200, CST), anti-Ki67 (1:200, CST).

### TUNEL assay

The slides were de-paraffinized, rehydrated, permeabilized, and applied to the TdT activity test with a TUNEL assay kit (Elabscience). Briefly, each slide sample was treated with 100 μL 1 × proteinase K working solution and incubated in 37 °C for 20 min. After rinsing three times with 1x PBS, the endogenous catalase activity was blocked by 3% hydrogen peroxide solution at room temperature for 10 min and rinsed 3 times with 1x PBS. 100 μL TdT equilibration buffer was added to each sample and incubated at 37 °C for 20 min, then 50 μL TdT working solution was added and the reaction was performed at 37 °C for 60 min in dark. Finally, 100 μL streptavidin-HRP working solution was added and incubated 30 min at 37 °C in dark. After rinsing 3 times with 1x PBS, DAB solution was applied for color development. The images were captured by a Leica DMLS microscope (Leica, Germany). About 5–6 images were collected for quantitative analysis by ImageJ (USA).

### BrdU incorporation assay

The mice were injected intraperitoneally with BrdU (Sangon Biotech, 200 µg), the tissues were harvested at 6, 24, and 72 h post BrdU injection and prepared for immunohistochemistry staining using BrdU kit (BD). Briefly, after blocking with 10% goat serum, the anti-BrdU antibody (1:9) was applied and incubated overnight at 4 °C. After rinsing 3 times with 1x PBS, the DAB solution was applied for color development. The images were captured by a Leica DMLS microscope (Leica, Germany). About 5–6 images were collected for quantitative analysis by ImageJ (USA).

### Alcian blue staining

The tissue sections were dewaxed and rehydrated, then stained with Alcian Blue kit (Solarbio). Briefly, the slides were stained with Alcian blue solution for 10 min. After washing with water, the 3% hydrogen peroxide was added and treated for 5 min. After washing with water, the Schiff staining solution was applied and stained for 15 min. After washing with water, the hematoxylin solution was applied for 30 s to stain the nuclei. After washing with water, the acid differentiation solution was applied for 2 s for differentiation. The images were captured by a Leica DMLS microscope (Leica, Germany). About 5–6 images were collected for quantitative analysis by ImageJ (USA).

### Isolation of intestine crypts

The mouse small intestine was cut lengthwise and washed 3 times in cold 1x PBS. After removed the villi with a glass slide, the small intestine was washed three times in cold 1x PBS and cut into chunks of about 1 cm and transferred to 5 mM EDTA for digestion at 4 °C for 40 min. The intestinal mass was removed, and the digestion solution was centrifuged at 800 rpm for 5 min. The precipitate was collected and re-suspended in cold 1x PBS and centrifuged at 600 rpm for 2 min. The precipitate was collected as isolated crypts for extracting crypt proteins and RNA.

### Immunofluorescence

The cells cultured on cover slips were fixed with 2% paraformaldehyde and 2% sucrose in 1x PBS for 20 min and then permeabilized with 1% NP-40. After pre-incubation with 5% BSA/PBS, cells were incubated first with the primary antibody overnight and then with the secondary antibody in 1% BSA/PBS for 1 h at room temperature. The slides were mounted with anti-fade mounting medium with DAPI (Solarbio). The images were captured by an Olympus SpinSR10 confocal microscope (Olympus, Japan). The primary antibodies used were anti-Hes1 (1:300, CST), anti-β-Catenin (non-phosphorylated (active), 1:300, CST), anti-Notch1 (1:300, CST), anti-Wnt3 (1:300, Abcam).

### qPCR

Total RNA from isolated crypts was isolated by Trizol followed by the RNeasy Mini kit (Qiagen) purification. The cDNA was reverse transcribed, and the quantitative PCR (qPCR) was performed on an ABI Prism 7300 sequence detection system with SYBR-Green PCR master mix according to the manufacturer’s instructions (Applied Biosystems). The primers used are as follows: Axin:Forward: TGCCCGTTTCCTCTAATGCT, Reverse:CCGTCCATCAAGCTTGCATT; Lgr5:Forward: CCTCAGCGTCTTCACCTC, Reverse:CATTTCCAGCAAGACGTAACT; Wnt3:Forward: CCGCAATTACATCGAGATC, Reverse:TGCACGAAGGCCGATTCAC; C-Myc:Forward: CGCGTCCGAGTGCATTG, Reverse:GGGCGGTGTCTCCTCATG; CD44:Forward: AAGAAGGGCGAGTATAGAACA, Reverse:TCTGGGGTGCTCTTCTCGA; Actin: Forward: CCTCACTGTCCACCTTCC, Reverse:GGGTGTAAAACGCAGCTC.

### Co-immunoprecipitation (Co-IP)

The WT, p21 null, G2DKO, and p21G2TKO MEF cells were harvested, and cell lysates were prepared by mild RIPA buffer (Beyotime) containing proteinase inhibitor cocktail (Roche). The protein concentration of cell lysates was measured and adjusted to same concentrations. The cell lysates were pre-clear with Protein A/G Magnetic Beads (Thermo) at 4°C for 2 h, the supernatants were collected and incubated with the same Protein A/G Magnetic Beads and primary antibodies at 4°C overnight. The precipitates were collected and applied to Western blotting analysis. The primary antibodies used are: anti-Lin9 (1:100, Proteintech), anti-β-Catenin (non-phosphorylated (active), 1:100, CST), anti-E2F1 (1:100, Novus).

### Chromatin Immunoprecipitation (ChIP) assay

The ChIP assay was performed with the Pierce Magnetic ChIP Kit (Thermo) according to the instruction of manufacturer. Briefly, the cell culture from the WT, p21 null, G2DKO, and p21G2TKO cells were cross-linked by 1% formaldehyde, and the cells were collected, and digested with lysis buffer contain 2 units of micrococcal nuclease, followed by sonication. After sonication, the supernatant was collected and incubated with ChIP grade protein A magnetic beads and anti-E2F1 antibody (Novus, used 2 µg for 20 µg of chromatin DNA), or anti-β-Catenin antibody (non-phosphorylated (active), CST, used 2 µg for 20 µg of chromatin DNA) overnight at 4 °C. The anti-IgG (Thermo, used 2 µg for 20 µg of chromatin DNA) was used as negative control, and anti-RNA Polymerase II antibody (Thermo, used 2 µg for 20 µg of chromatin DNA) was used as positive control. The beads were then washed and eluted. Twenty microliters of 5 M NaCl was added to the eluted product and incubated at 65 °C for 1.5 h to reverse the crosslinking. Immunoprecipitated genomic DNA was then purified and analyzed by qPCR. The primers for qPCR were: Notch1: Forward: ACTGACCCCATGCTGCTAAC, Reverse: CCCATCTCCCTTGACACACC; Hes1: Forward: GCGTGTCTCTTCCTCCCATT, Reverse: TCCAGGACCAAGGAGAGAGG;

### Flow cytometry assay

The WT, p21 null, G2DKO, and p21G2TKO MEF cells were harvested and fixed in ice-cold 70% ethanol overnight. The cells were then washed with 1x PBS and stained with 1x PBS based propidium iodide solution (50 µg/ml PI, 100 µg/ml RNase A, 0.1% sodium citrate,0.1% Triton X-100) at 4 °C overnight in dark. The stained cells were applied to flow cytometry analyses (Agilent, USA).

### Overexpression of p21

The p21 expression construct p21-pQXCIP was constructed previously [43]. Briefly, the p21 cDNA sequence was amplified from wild type MEFs and cloned into pQXCIP plasmid. The construct and the p21 sequence were confirmed by DNA sequencing. The p21-pQXCIP and the retrovirus packaging plasmid pCL-Eco were transfected (Lipofectamine 3000, Invitrogen) and packed in 293 T cells. The supernatant of transfected 293 T cells were collected and used to infect the p21G2TKO cells. After 48 h of infection, 1 µg/ml Puromycin (Meilunbio) was added to the culture medium to select the cells with p21 overexpression. After 48 h, the p21G2TKO cells were collected and used for experiments.

### Quantification and statistical analysis

All statistical data were processed and analyzed with Graphpad prism (Version 5, http://www.graphpad-prism.cn/) by unpaired two-sided *t*-test. All experiments were repeated at least three times (*N* = 3, biological replicates). The results in figures were presented as mean ± SEM. Statistical significance is indicated with asterisks as follows: ns, no significance; *, *p* < 0.05; **, *p* < 0.01; ***, *p* < 0.001; ****, *p* < 0.0001.

## Supplementary information

supplementary materials

## Data Availability

The datasets have been deposited in GEO database, the materials used in this paper will be available upon request.
