# Supplementary material for: p21 Regulates Wnt-Notch balance via DREAM/MMB/Rb-E2F1 and maintains intestinal stem cell homeostasis
Source: Cell Death Discov. 2024 Sep 28;10:413. doi: 10.1038/s41420-024-02192-z (PMC11438959; doi:10.1038/s41420-024-02192-z)

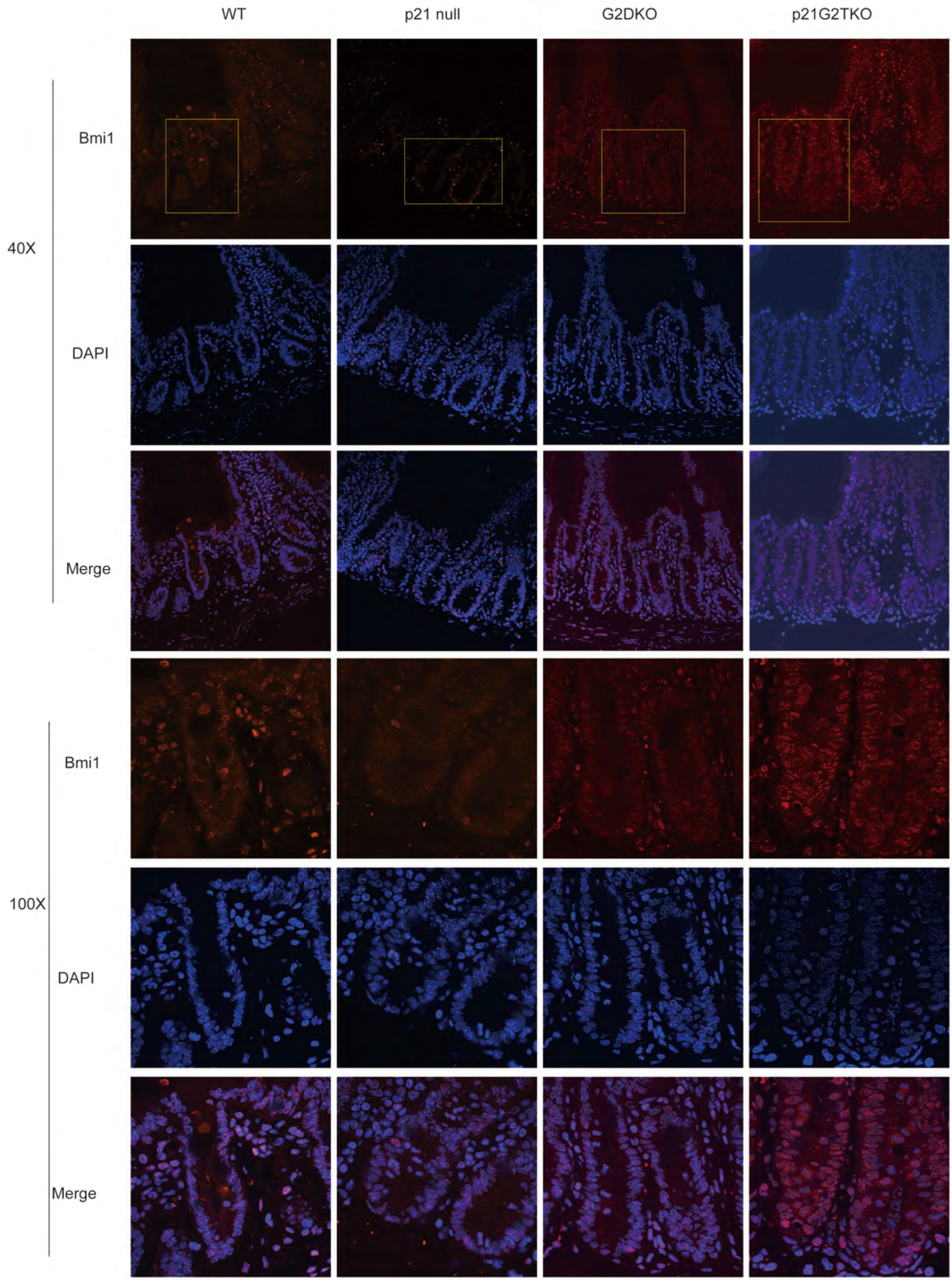

Supplementary Figure 1. The immunofluorescence staining of Bmi1 confirmed the increase of Bmi1+ cells in the crypts of p21G2TKO mice. The upper panel images are taken with 40x magnification and the frames indicate the areas enlarged in 100x magnification are shown in the lower panel images.

Figure 2B   original WB blots

Wnt3

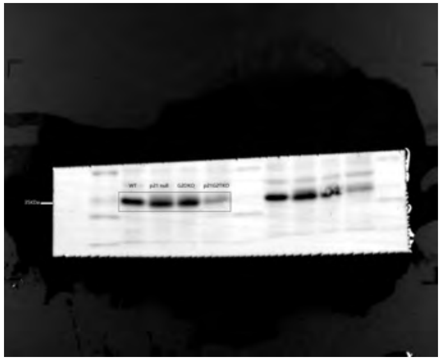

Notch1

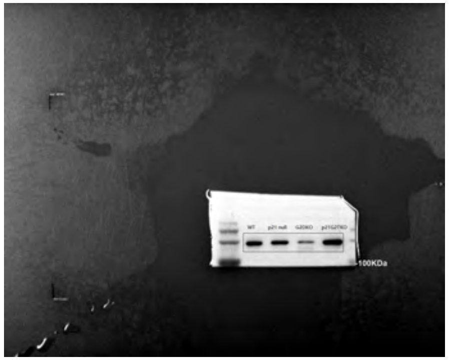

Hes1

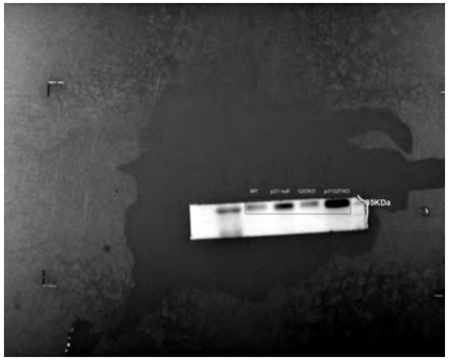

Olfm4

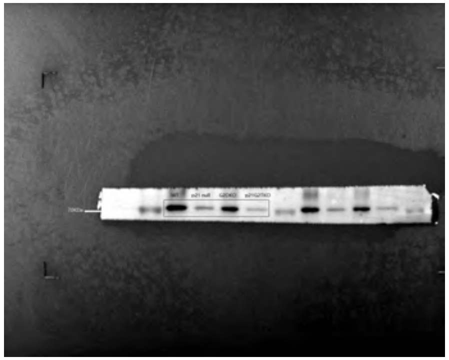

Lysozyme

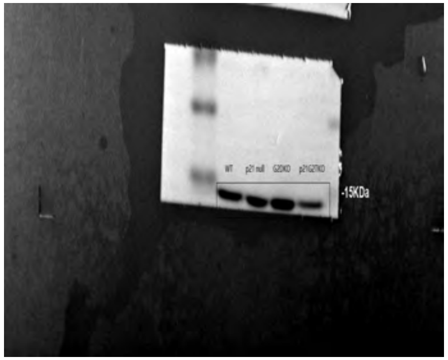

β-Actin

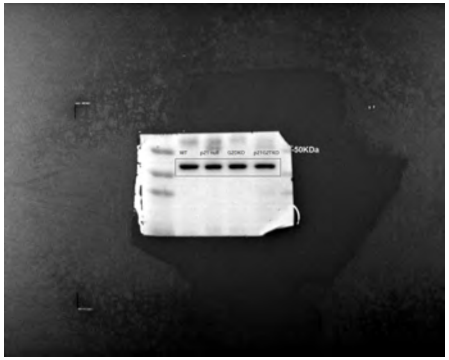

Figure 2C original WB blots

Wnt3

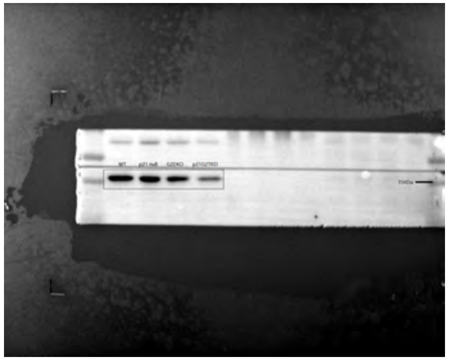

B-Catenin

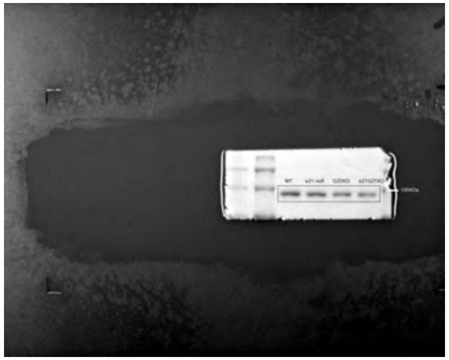

B-Catenin

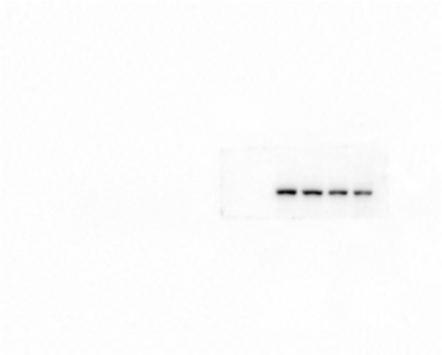

Notch1

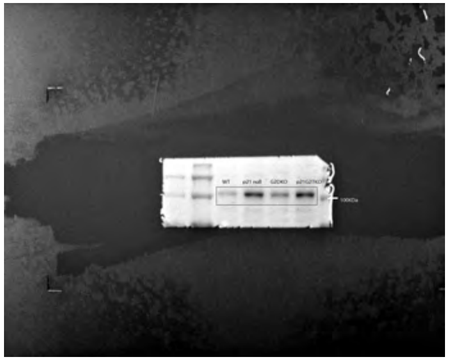

Hes1

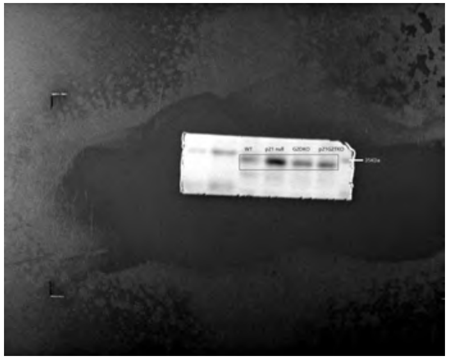

Rbpj

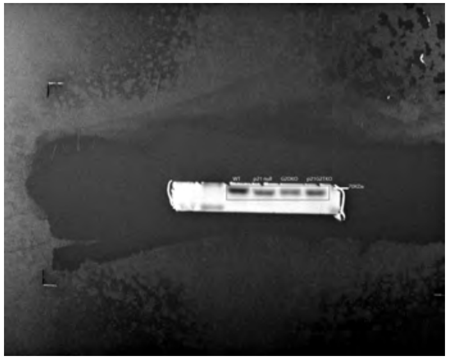

Rbpj

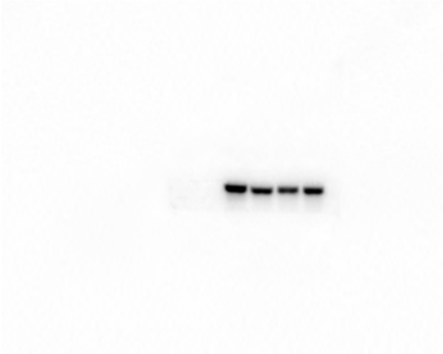

β-Actin

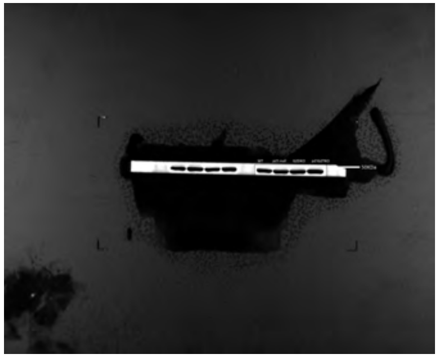

Figure 3E original WB blots

p-H3

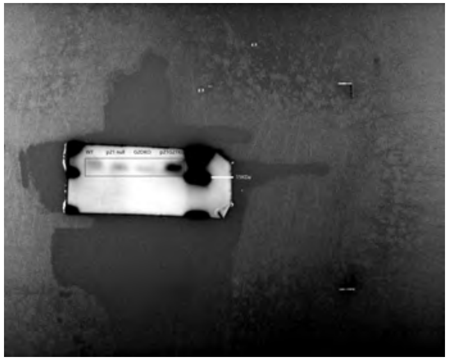

Bmi1

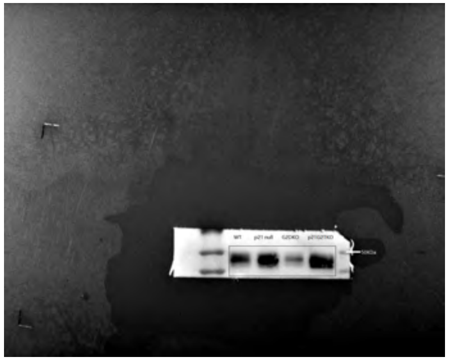

PCNA

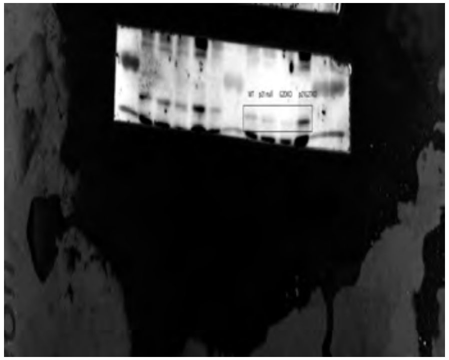

β-Actin

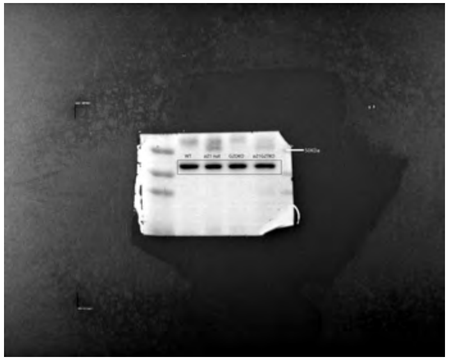

Figure 3F original WB blots

Lin9

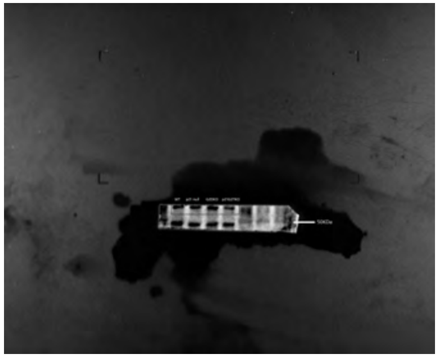

Lin54

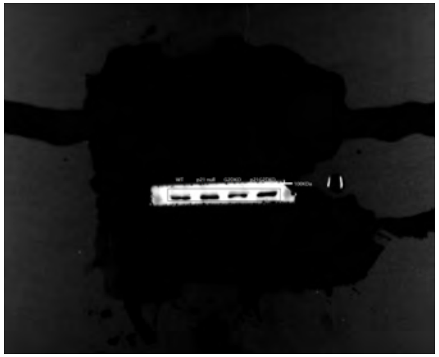

B-Myb

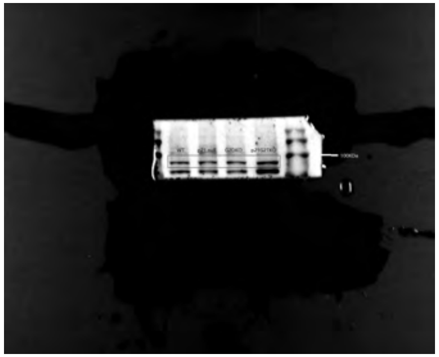

P130

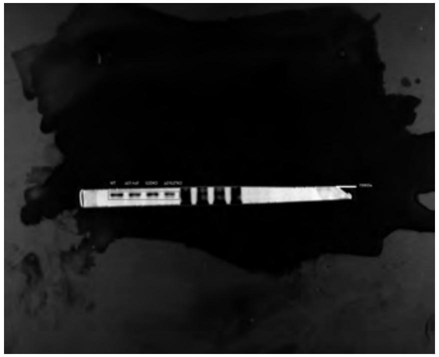

P107

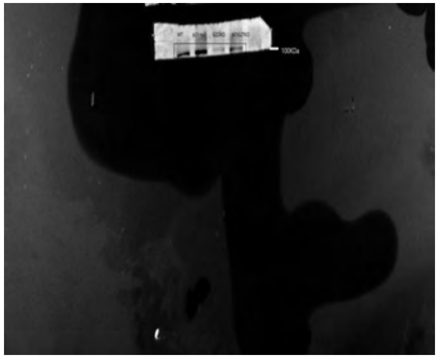

Rb

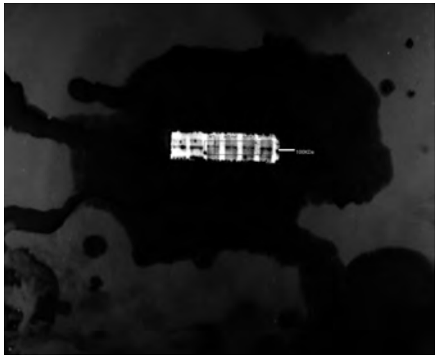

E2F1

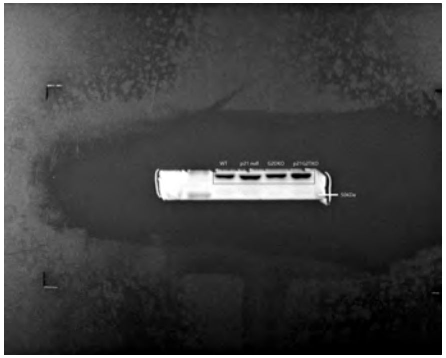

Mcm2

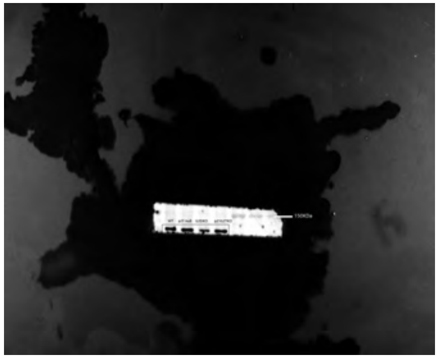

Figure 3F original WB blots

Mcm7

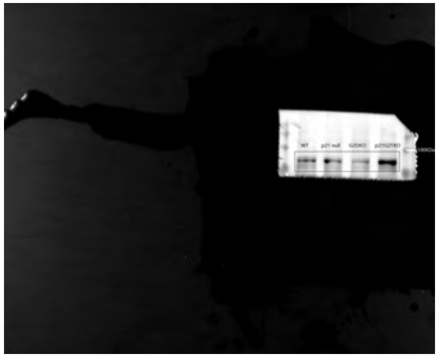

Suvivin

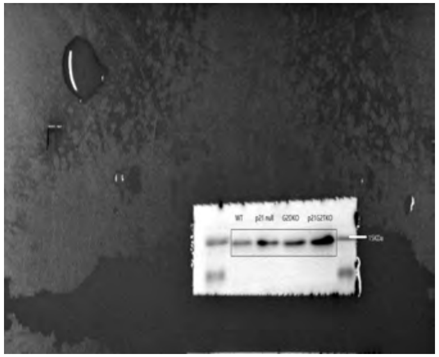

p21

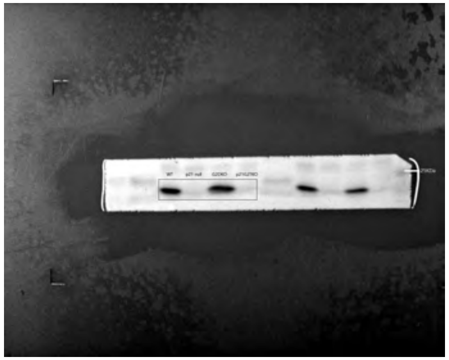

p53

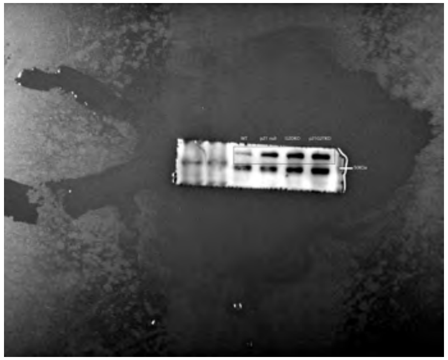

β-Actin

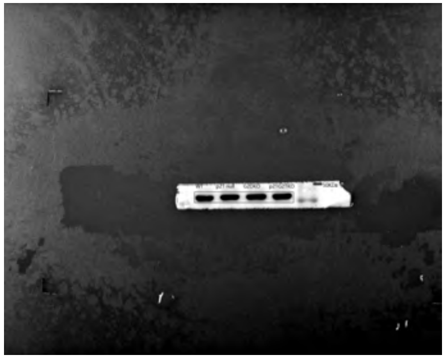

Figure 3G original WB blots

Cdc2

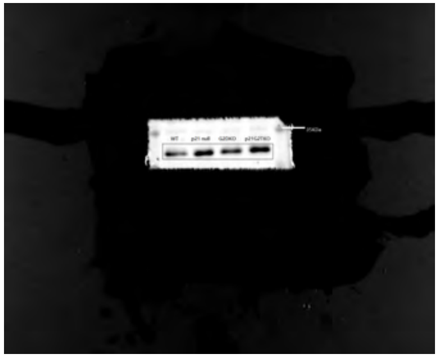

Cdk2

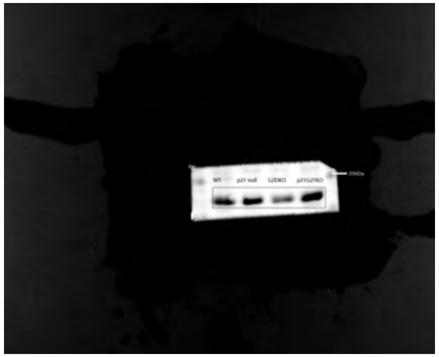

Cdk4

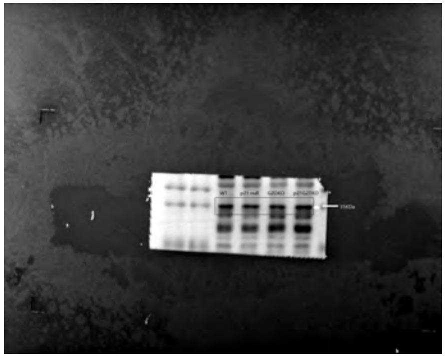

Cdk6

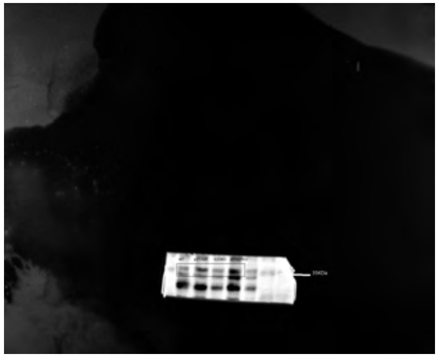

Cdc25c

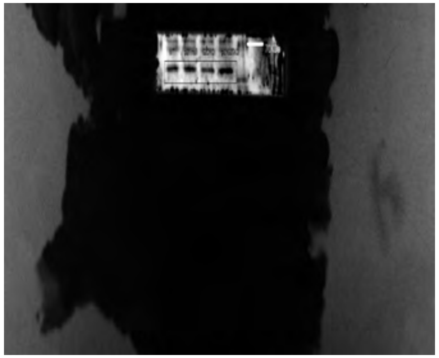

Wee1

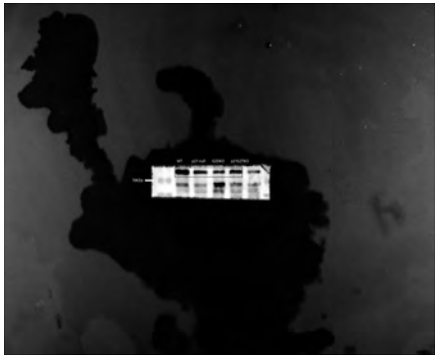

p-Cdc2

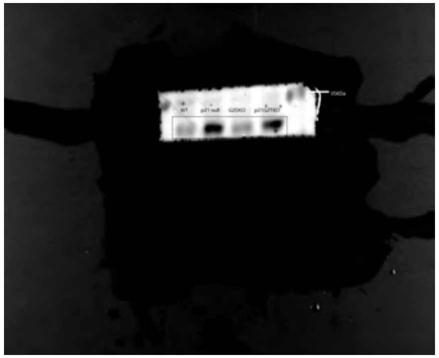

Cyclin B1

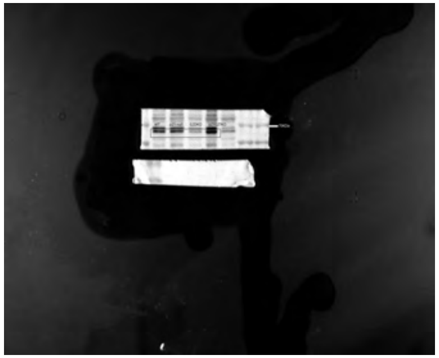

Figure 3G original WB blots

p-H3

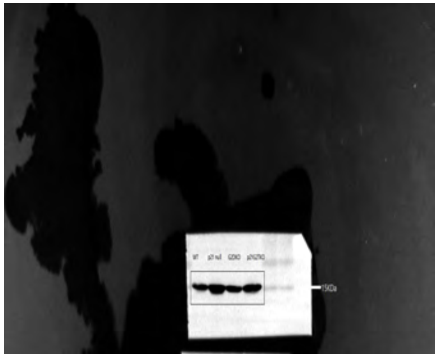

Bmi1

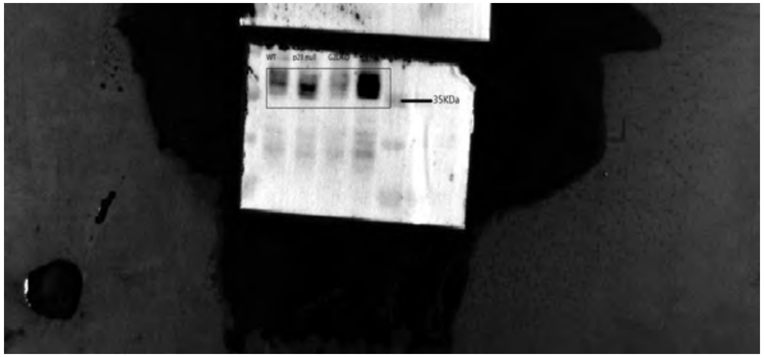

PCNA

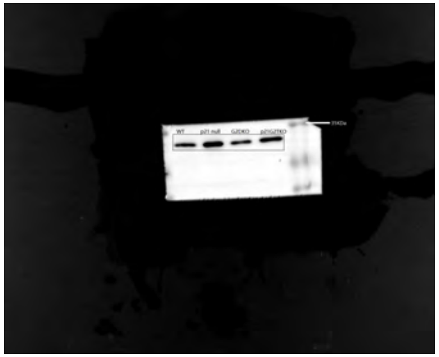

Ki67

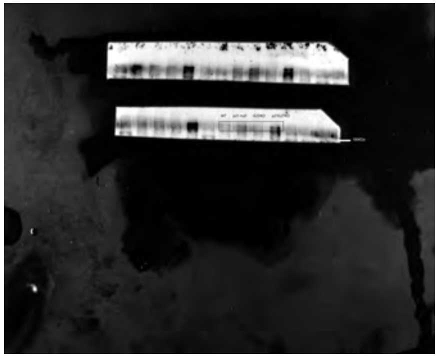

β-Actin

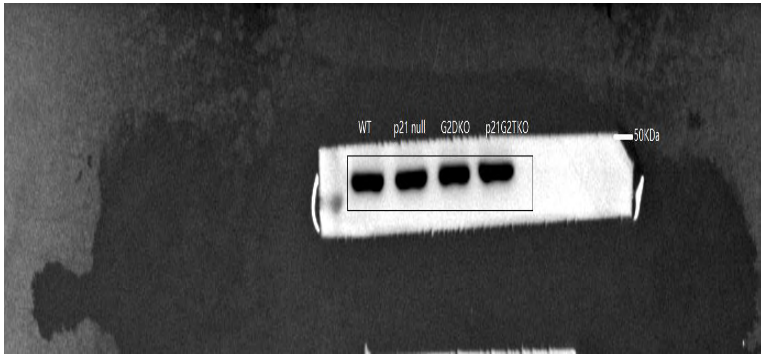

Figure 3H original WB blots

Lin9

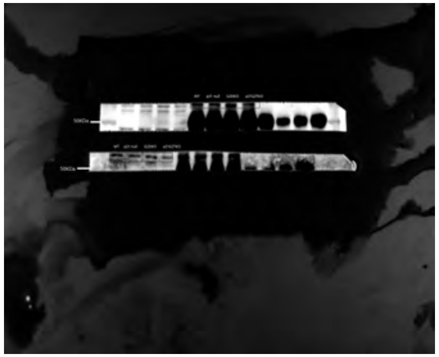

B-Myb

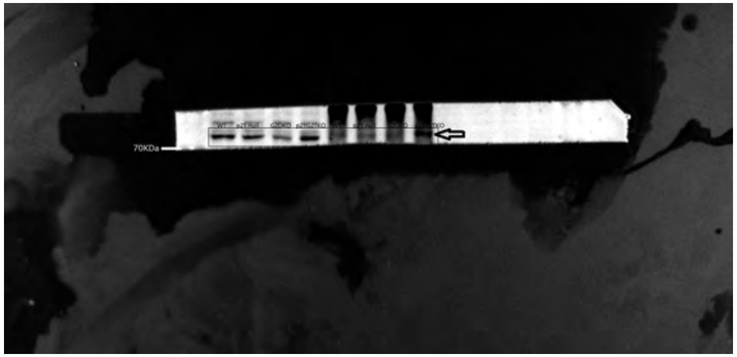

P130

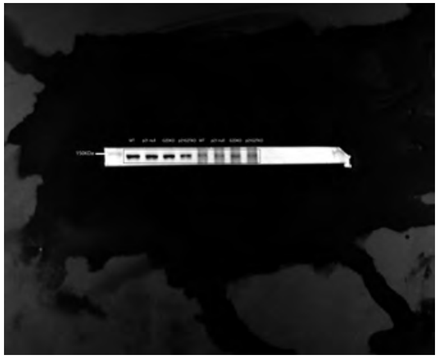

GAPDH

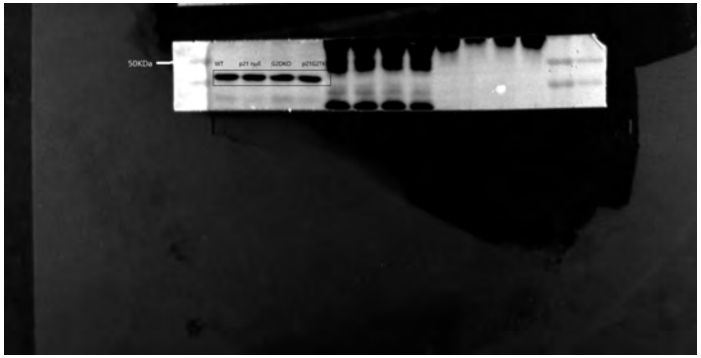

Figure 4A original WB blots

E2F1-Input

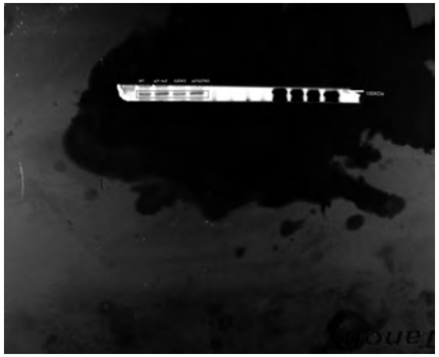

E2F1-E2F1

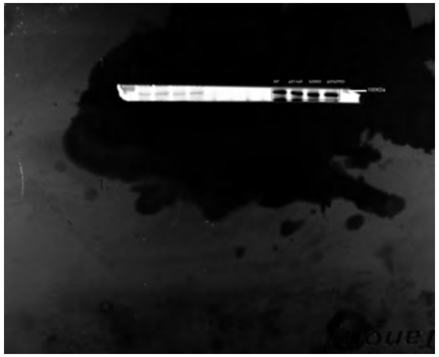

P107-Input

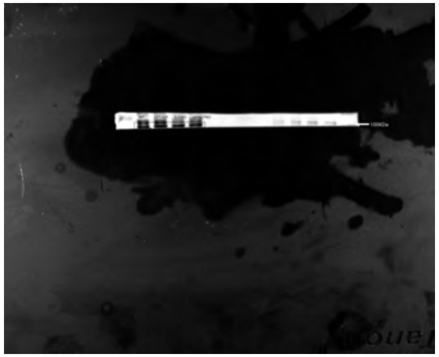

P107-E2F1

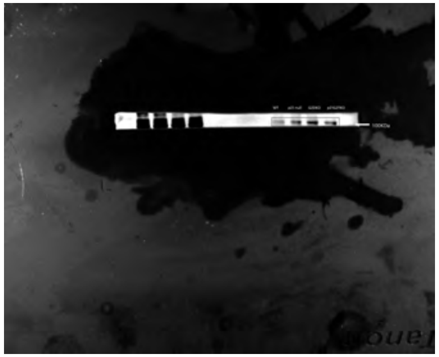

P130

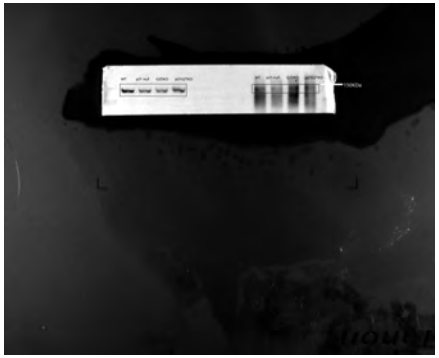

Rb-Input

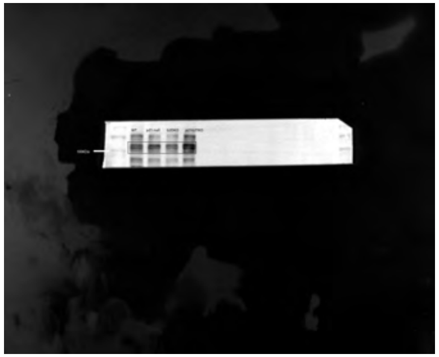

Rb-E2F1

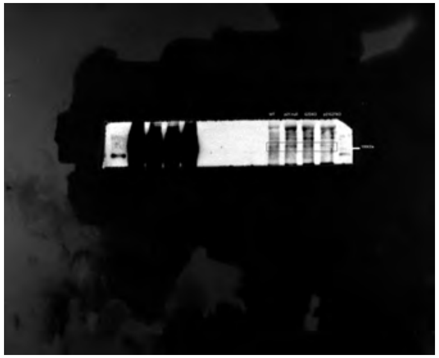

GAPDH

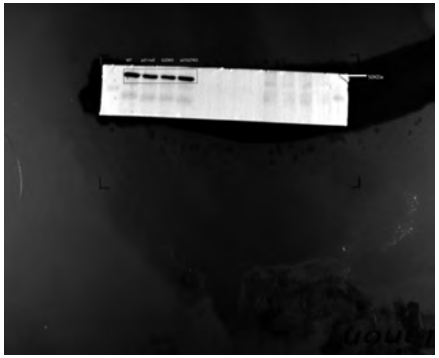

Figure 4C   original WB blots

B-Catenin

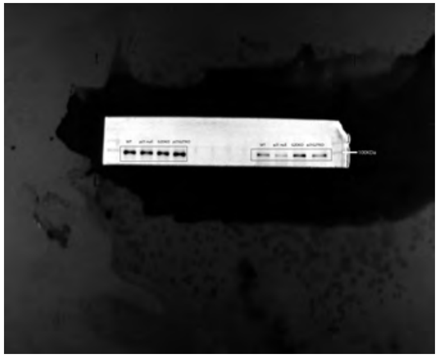

Notch1-Input

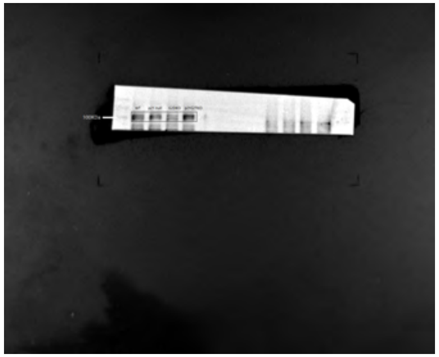

Notch1-B-Catenin

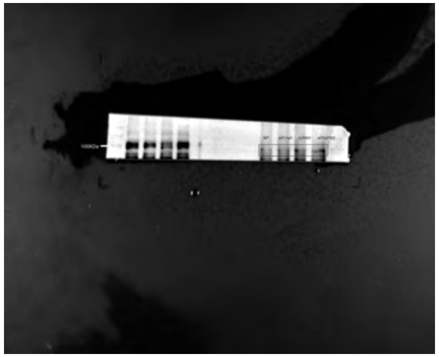

Rbpj

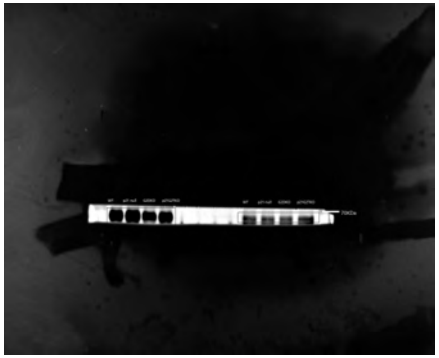

H2B

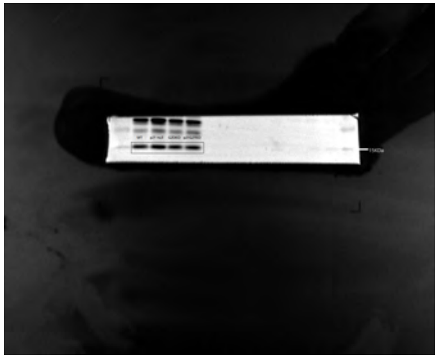

Figure 5A original WB blots

p21

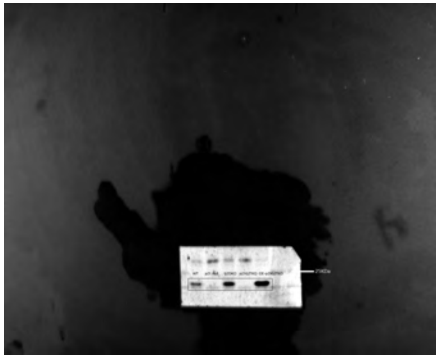

Wnt3

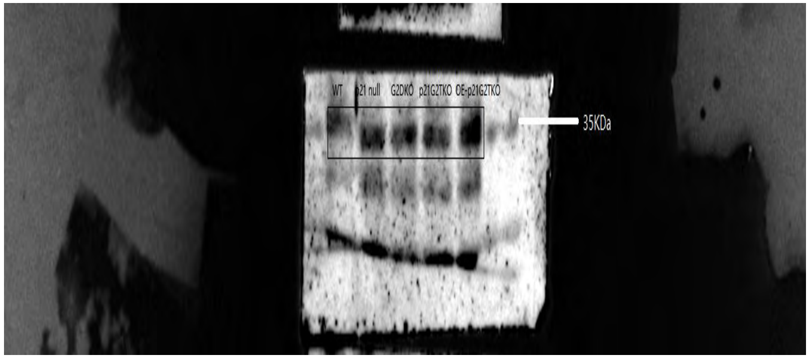

B-Catenin

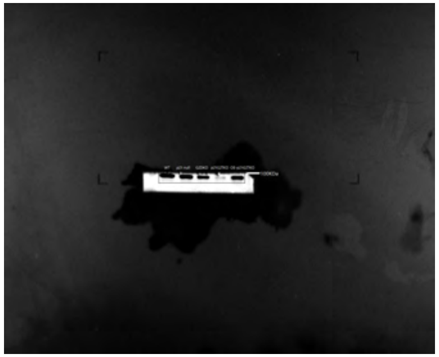

Notch1

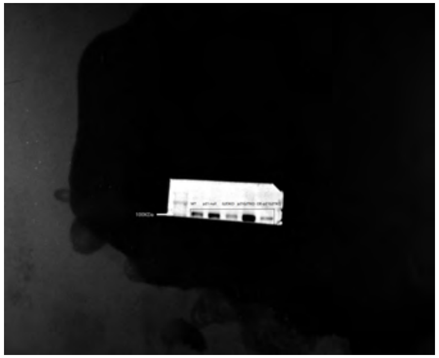

Hes1

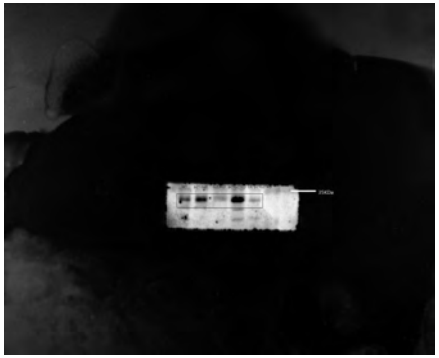

Rbpj

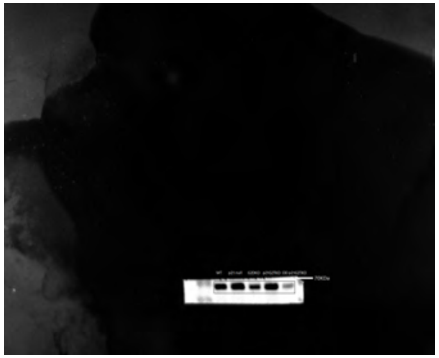

β-Actin

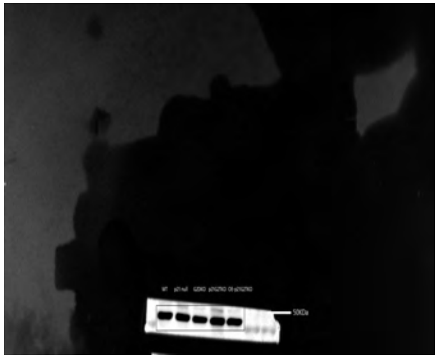

Figure 5B   original WB blots

Lin54

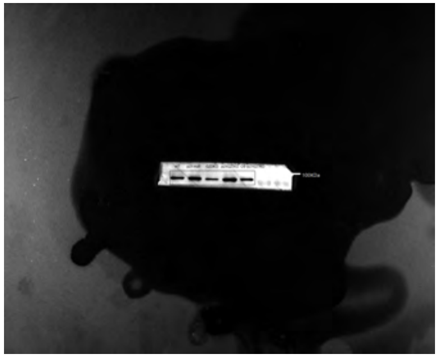

B-Myb

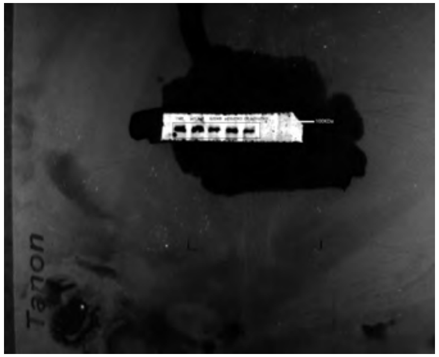

P130

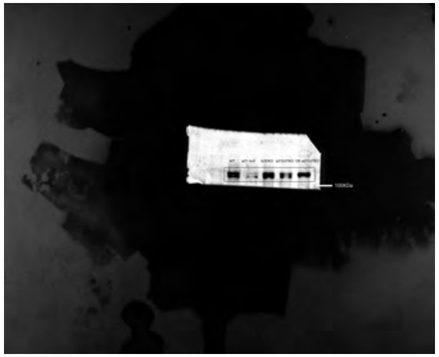

P107

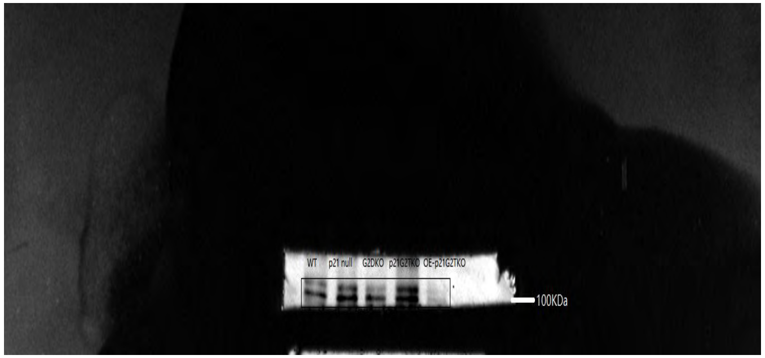

Rb

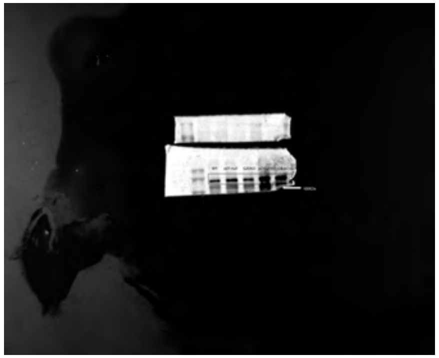

E2F1

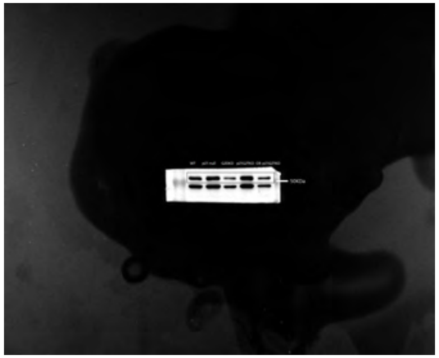

Figure 5B   original WB blots

Mcm2

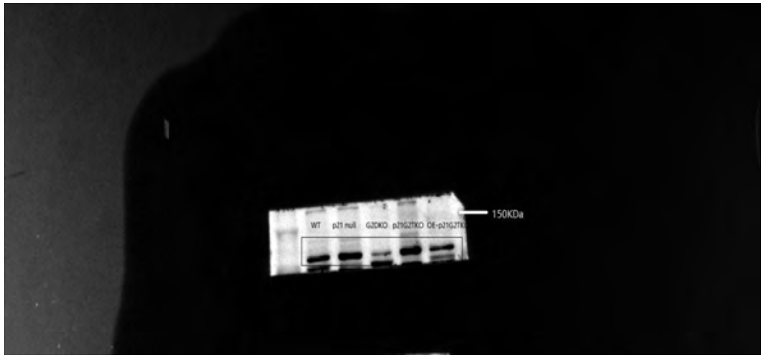

Mcm7

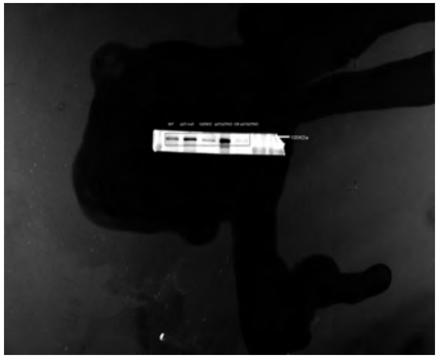

Suvivin

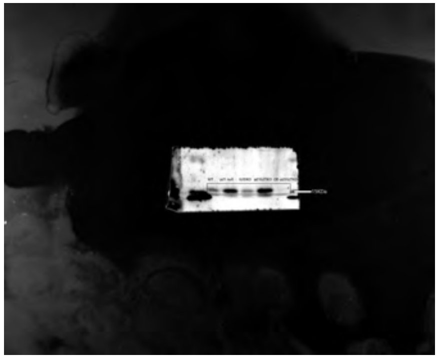

p53

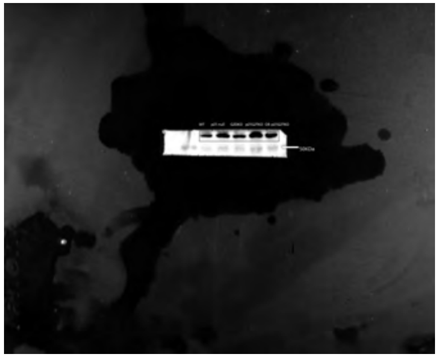

β-Actin

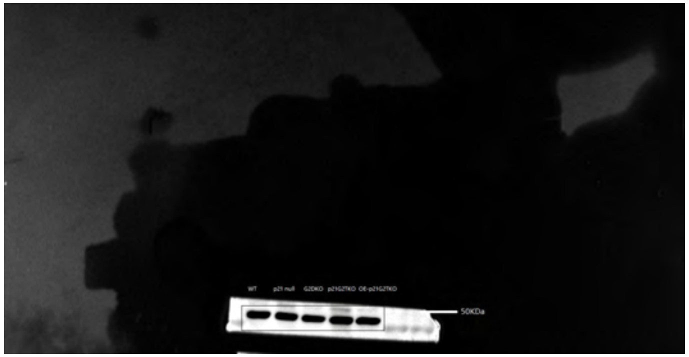

Figure 5C original WB blots

Cdc2

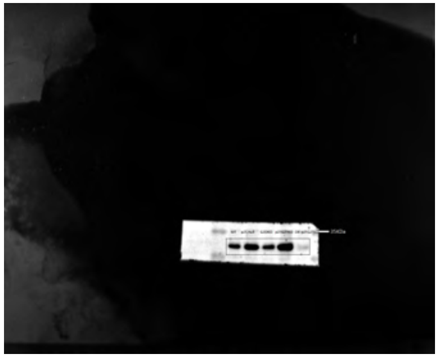

Cdk2

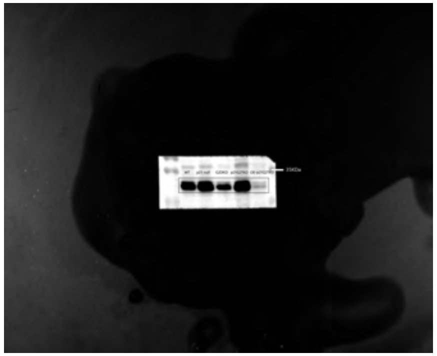

Cdk4

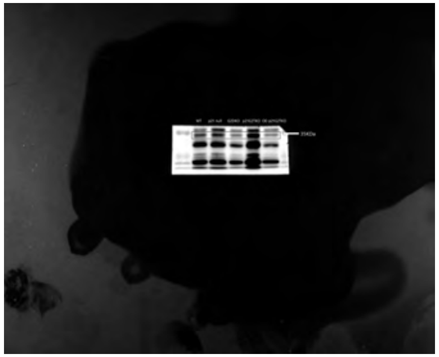

Cdk6

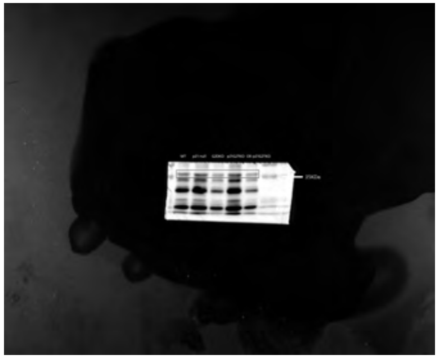

Cdc25c

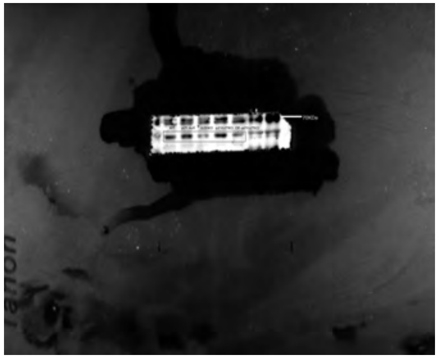

Wee1

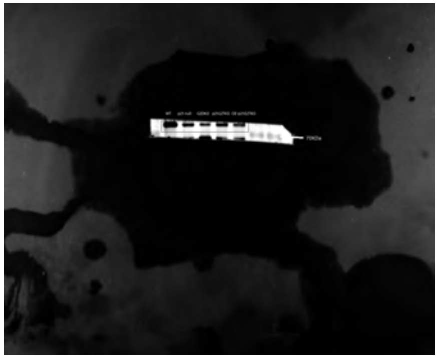

p-Cdc2

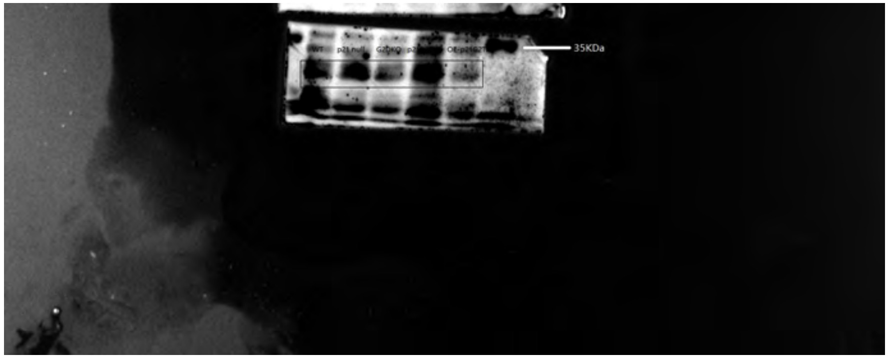

Figure 5C original WB blots

Cyclin B1

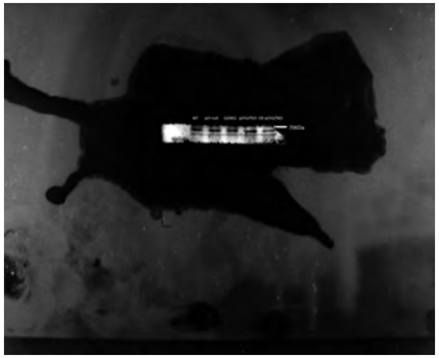

p-H3

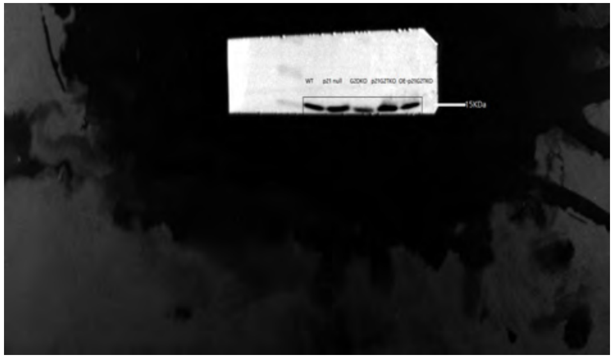

Bmi1

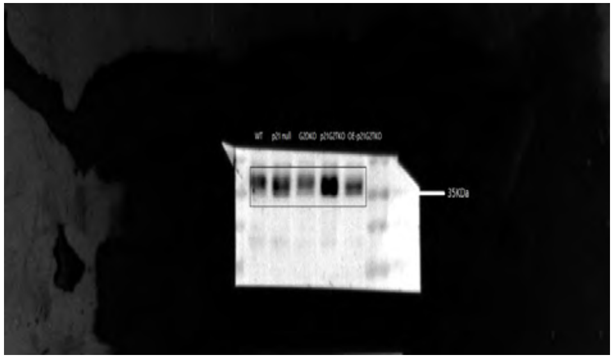

PCNA

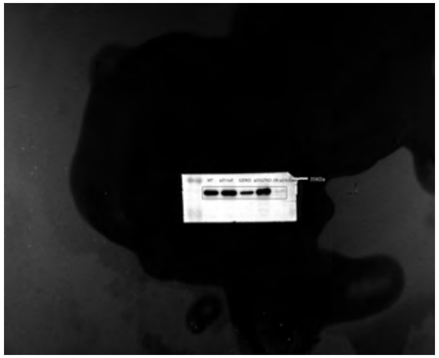

Ki67

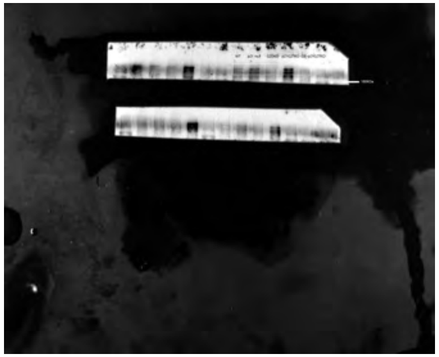

β-Actin

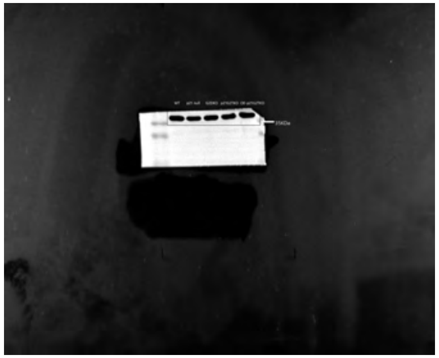

Supplement: Supplementary file 1 — supplementary materials [file 41420_2024_2192_MOESM1_ESM.pdf]
